# Whole-Genome Sequencing Revealed a Late-Maturing Isogenic Rice Koshihikari Integrated with *Hd16* Gene Derived from an Ise Shrine Mutant

**DOI:** 10.1155/2022/4565977

**Published:** 2022-01-06

**Authors:** Motonori Tomita, Ryotaro Tokuyama, Shosuke Matsumoto, Kazuo Ishii

**Affiliations:** ^1^Research Institute of Green Science and Technology, Shizuoka University, 836 Ohya, Suruga-ku, Shizuoka City, Shizuoka 422-8529, Japan; ^2^Faculty of Agriculture, Tottori University, 4-101 Koyama Minami, Tottori City, Tottori 680-8550, Japan; ^3^Faculty of Engineering, Suwa University of Science, 5000-1 Toyohira, Chino City, Nagano 391-0292, Japan

## Abstract

We identified the key genes controlling the late maturation of the Japonica cultivar Isehikari, which was found at Ise Jingu Shrine and matures 6 days later than Koshihikari. We conducted a genetics-based approach through this study. First, the latest mature plants, which flowered later than Isehikari, were segregated in the F_2_ and F_3_ generations of Koshihikari×Isehikari. Next, the linkage relationship of a single late-maturing gene with the SSR markers on the long arm of chromosome 3 was inferred by using late-maturing homozygous F_2_ segregants. Moreover, genetic analyses of late maturity were conducted through the process of six times of continuous backcross with Koshihikari as a recurrent parent by using the late-maturing homozygous F_3_ line as a nonrecurrent parent, thus developing a late-maturing isogenic Koshihikari (BC_6_F_2_). As a result, we elucidated a single late-maturing gene with incomplete dominance that caused the 14-day maturation delay of Koshihikari. The whole-genome sequencing was conducted on both of Koshihikari and the late-maturing isogenic Koshihikari. Then, the SNP call was conducted as the reference genome of Koshihikari. Finally, a single SNP was identified in the key gene *Hd16* of the late-maturing isogenic Koshihikari.

## 1. Introduction

Rice is cultivated worldwide, particularly in Asia, and is one of the world's top three grains with an annual yield of 680 million tons, alongside corn (1.73 billion tons) and wheat (680 million tons) [1]; therefore, stable production is crucial. The earth has warmed by approximately 1.0°C from preindustrial levels, and temperatures are predicted to rise another 1.5°C between 2030 and 2052 [2]. In the IPCC's 6^th^ evaluation report, global warming is expected to bring about an increase in the frequency of strong tropical cyclones, and there are concerns that damage from heavy rains will be magnified [3, 4]. Rice has suffered from lodging damage, yield reduction, lowered grain quality by ear germination, and lowered production efficiency, which are brought by serious strong winds and rain [5]. Furthermore, if the average daily temperature exceeds 23°C–24°C during the 20 days after heading, a white immature grain arises [6]. The white immature grain occurs when warm nights lead to increased respiration, causing a reduction in nitrogen content, and consequently reducing the transportation of photosynthetic products (sugars), which serve as a substrate for starch, to the panicles; thus, it results in an imbalance in nutrient supply and demand [7–9]. At 27°C, both white-back immature grains and milky-white immature grains arise; at 30°C, white-back immature grains occur frequently; and at 33°C, milky-white immature grains frequently happen [10]. Climate changes over recent years have resulted in record-breaking heat waves, with a spread in the deterioration of rice quality. In 2011, 170,000 tons of rice, or 21% of the total production volume, suffered high-temperature damage [11]. The proportion of 1^st^-grade quality rice has been greatly reduced over the last 4 years from 78.8%–85.7% to 62.4% [12]. This is because the leading variety Koshihikari, which comprises 37.3% of rice acreage in Japan [13], heads and ripens in the high-temperature phase in August. In 2010, when the average temperature in August was approximately 2.25°C higher than the yearly average, the grain quality noticeably degraded, and the proportion of 1^st^-grade rice was 23.1% lower than in 2009 [14]. In 2018, disastrous rainfall represented as “Heavy Rain in July, Heisei 30”, large typhoons, and heat wave over 40°C caused poor filling and widespread yield reduction in Japan [15–19]. Koshihikari is globally valued and produced including in the United States and Australia. To continue the commercial trend of Koshihikari, it is necessary to avoid heading and ripening during the high temperature phase by genetically altering rice maturation to the early or late phase. Rice production industries also request late-maturing varieties instead of Koshihikari to avoid high temperature ripening. Modifications in day length responsiveness enable selecting regionally adaptive new genotypes and dispersing the current overconcentration on Koshihikari.

Isehikari was discovered in 1989 in Ise Jingu Shrine in Ise City in Mie Prefecture, when a typhoon caused the large-scale lodging of Koshihikari in the shrine's rice fields, and only Isehikari remained standing [20, 21]. This cultivar can withstand typhoon wind speeds exceeding 50 m/s, and it matures 10 days later than Koshihikari [22, 23]. In terms of yield, Isehikari was used in Yamaguchi Prefecture's Yamaguchi Active Aging City “Environmental Future City Concept,” in which 70% of farmers gained a yield of over 500 kg per 10 acres and heavily fertilized areas displayed a potential high yield of 700 kg [24]. Thus, Isehikari is an extremely beneficial genetic resource; however, its nature of genetic alteration is unclear. Therefore, molecular genetic analyses for useful variations such as late maturation and short culms are extremely essential for efficient application in practical rice breeding.

In this study, we focused on the late-maturation trait of “Isehikari,” and we conducted a genetics-based genomics approach. We firstly conducted genetic analyses for late maturity in F_2_ and F_3_ between Koshihikari×Isehikari. Because this is a hybrid between Japonica cultivars, there are only 66 SSR markers, which show DNA polymorphisms between Japonica cultivars. As polymorphic DNA markers were limited, first, we roughly mapped the late-maturation gene in the 30–33 Mb region of chromosome 3 by using polymorphic F_2_ generations. We then proceeded six times of continuous backcrossing with Koshihikari to develop late-maturing isogenic Koshihikari, which was introduced with the late-maturing gene from Isehikari. Then, we conducted a whole-genome sequencing analysis of the late-maturing Koshihikari, and finally, we identified only a single SNP in *Hd16* among the coding sequences in the 30–33 Mb region of chromosome 3. The developed 14-day late-maturing Koshihikari with *Hd16* was registered as a new cultivar “Koshihikari Suruga Hd16” under Japanese varietal protection, which is useful to avoid flowering and maturity in the hottest August.

## 2. Materials and Methods

### 2.1. Genetic Analysis and Development of Late-Maturing Isogenic Line

First, a genetic analysis on heading date was conducted by using 149 individuals in the F_2_ of Koshihikari×Isehikari. Because the genetic background between Koshihikari and Isehikari was divergent, heading-date distribution in the F_2_ of Koshihikari×Isehikari was continuous, and late-maturing individuals that matured 7 days later than Isehikari were segregated. Then, progeny tests were conducted on 70 randomly selected F_2_ plants by using self-propagated 70 F_3_ lines consisting of 570 individuals derived from each 70 F_2_ individuals to identify the genetic mode of heading date. The self-propagated F_3_ lines were cultivated within the Field Science Center's fields, and the heading date, culm length, and phenotype were examined. Late-maturing homozygote in the F_3_ of Koshihikari×Isehikari was utilized as a nonrecurrent parent; six times of continuous backcross with Koshihikari as a recurrent parent were conducted. Finally, the late-maturing isogenic Koshihikari, Koshihikari×6/[(Koshihikari×Isehikari) F_2_ late-maturing type] BC_6_F_2_, was developed. In each BC*_n_*F_2_ generation through the backcrossing process, the heading date was genetically analyzed, and the latest mature segregants were isolated and used as pollen parents for the backcross with Koshihikari as a recurrent parent.

Cultivation of genetic materials was carried out in a paddy field at Shizuoka University, Shizuoka, Japan, from 2013 to 2019. Genetic materials were sown in late June, and then, seedlings were individually transplanted into a paddy field in mid-July with a transplanting density of 22.2 seedlings/m^2^ (one seedling per 30 × 15 cm). The paddy field was fertilized by 4.0 kg of basal fertilizer containing nitrogen, phosphorus, and potassium (weight ratio, nitrogen : phosphorus : potassium = 2.6 : 3.2 : 2.6) at a rate of 4.3 g/m^2^ nitrogen, 5.3 g/m^2^ phosphorus, and 4.3 g/m^2^ potassium across the field. The heading date was recorded as the date the first panicle had emerged from the flag leaf sheath for each plant. Culm length was measured as the length between the ground surface and the panicle base.

### 2.2. Mapping of Late-Maturation Gene by DNA Markers

In the F_3_ progeny test, 23 F_3_ lines were fixed in late-maturation headings between 8/31 and 9/8, which derived from late-maturing F_2_ plants that head from 8/23–9/2. Namely, the progeny from late-maturing F_2_ plants was confirmed to be fixed to late-maturating homozygotes. The late homozygous 23 F_2_ plants fixed in the F3 were used to roughly map a late-maturation gene linkage by using 66 SSR markers. F_2_ leaves were placed in a 2 mL tube with ceramic beads, immersed in liquid nitrogen and frozen, and pulverized with a high-speed bead mill homogenizer, the Precellys 24 high-throughput bead-mill homogenizer (Bertin Technologies, Montigny-le-Bretonneux, France), by 6500 rotations with two periods of 20 s with a 5 s interval. Genome DNA was extracted by using the cetyltrimethylammonium bromide (CTAB) method [25]. Utilizing the 66 SSR markers that show DNA polymorphism between Koshihikari and Nipponbare, which dispersed on 12 rice chromosomes, a linkage analysis was conducted with a late-maturation gene. For SSR markers, using 20 ng each of rice genomic DNA as a template, 50 *μ*L of a reaction solution containing 200 nmol/L each primer (33 ng), 100 *μ*mol/L dNTPs, 50 mmol/L KCl, 10 mmol/L Tris-HCl (pH 8.8), 1.5 mmol/L MgCl_2_, and 1 U TaKaRa LA Taq (Takara Bio Inc., Kyoto, Japan) was prepared. Using the Thermal Cycler CFX96 (Bio-Rad Laboratories, Hercules, CA), the reaction solution was subjected to 35 cycles of denaturation at 94°C for 30 s, annealing at 55°C for 30 s, and primer extension at 72°C for 1 min. The first denaturation at 94°C and the last extension at 72°C were set for 5 min. The SSR polymorphisms in the PCR products were analyzed by electrophoresis using a cartridge QIAxcel DNA Screening Kit (2400) in a QIAxcel electrophoresis apparatus (QIAGEN, Hilden, Germany) at 5 kV for 10 min. Similarly, late-maturing homozygous segregants isolated from BC_3_F_2_ were used for mapping.

Late-maturing homozygous segregants in BC_5_F_2_ and BC_6_F_2_ were also confirmed by SSR marker RM16089 or TaqMan probe both tightly linked to SNP in *Hd16* identified by NGS. TaqMan probes specific to chromosome 3's SNP alleles in *Hd16* were designed as 5′-CTAGCGTATCTAATTGTTCCCCTGTAA-3′ for *hd16* in Koshihikari and 5′-CTAGTGTATCTAATTGTTCCCCTGTAA-3′ for *Hd16* in late-maturing isogenic Koshihikari (Isehikari) and labeled with florescent dyes FAM or HEX, respectively. The forward and reverse primers for the real-time PCR were 5′-TGGGCAATTATTAACTTACC-3′ and 5′-CTACCTGTACGACCTAAG-3′, respectively. The real-time PCR reaction was conducted to amplify the allele-specific fluorescence, by first heating the material to 95°C for 30 seconds to denature the DNA, then submitting it to 40 cycles of denaturing at 95°C for 15 seconds, and annealing at 48°C for 30 seconds.

### 2.3. Next-Generation Sequencing (NGS) Analysis

Whole-genome sequencing was conducted on both Koshihikari and the late-maturing homozygous isogenic lines (BC_4_F_2_, BC_6_F_2_), which were integrated with a late-maturing gene derived from Isehikari through continuous backcrossing into the genetic background of Koshihikari. The leaves were powdered using a mortar and pestle while being frozen in liquid nitrogen. The genomic DNA was then extracted from each cultivar by the CTAB method. Genomic DNA was fragmented and simultaneously tagged so that the peak size of the fragments was approximately 500 bp using the Nextera® transposome (Illumina Inc., San Diego, CA). After purification of the transposome by DNA Clean & Concentrator™-5 (Zymo Research, Irvine, CA), adaptor sequences, including the sequencing primers for fixation on the flow cell, were synthesized at both ends of each DNA fragment via a limited time of polymerase chain reaction, and then, size selection of DNA fragments was conducted by using AMPure XP magnetic beads (Beckman Coulter, Brea, CA). Finally, to prepare a DNA library for NGS, qualitative check by using a Fragment Analyzer™ (Advanced Analytical Technologies, Heidelberg, Germany) and quantitative measurements by a Qubit® 2.0 Fluorometer (Life Technologies; Thermo Fisher Scientific, Inc., Waltham, MA) were conducted. Sequencing was conducted by using the prepared DNA library in paired-end 2x 100 bp on a HiSeq 2500 next-gen sequencer, according to the manufacturer's protocols (Illumina Inc., San Diego, CA). The sequencing data were gained with paired-end reads. The gained Illumina reads were trimmed using Trimmomatic (version 0.39) [26]. The sequencing adapters and sequences with low quality scores on 3′ ends (Phred score [*Q*], <20) were trimmed. Raw Illumina WGS reads were quality checked by performing a quality control with FastQC (version 0.11.9; Babraham Institute). Mapping of reads from Koshihikari to the Nipponbare genome as a reference was conducted with Burrows-Wheeler Aligner (BWA) software (version bwa-0.7.17.tar.bz2) [27], duplicated reads were removed using Picard (version 2.25.5) (http://broadinstitute.github.io/picard), and secondary aligned reads were removed by using SAMtools (version 1.10.2) [28], to construct the consensus sequence of the Koshihikari genome. Next, the read sequences obtained from the late-maturing isogenic Koshihikari line were mapped by using the “consensus genome” of Koshihikari as a reference. To identify genetic variations among strains, single nucleotide variant (SNV) detection (variant calling) and SNV matrix generation were conducted using GATK version 4.1.7.0 [29].

## 3. Results

### 3.1. Inheritance and Phenotypic Expression of a Late-Maturing Gene in an Isogenic Background

**Figure 1:**
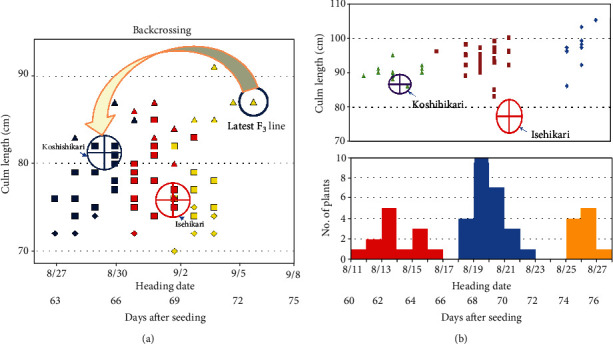
Genetic analysis for late maturity derived from Isehikari. (a) Distribution of mean value of heading date and culm length in 70 F_3_ lines derived from the cross Koshihikari×Isehikari. Progeny tests were conducted on 70 randomly selected F_2_ plants by using self-propagated 70 F_3_ lines from each F_2_ plant. The genotype of the heading date was determined according to the ratio of 23 late-maturing homozygous lines (tentatively *Hd* homo; blue) : 26 heterozygous lines (*Hdhd*; red) : 21 early maturing homozygous lines (*hd* homo; yellow), which fit to a theoretical single gene ratio of 1 : 2 : 1 (*χ*^2^ = 4.74, 0.05 < *p* < 0.10). The latest homozygous F_3_ line, which was 7 days later than Isehikari, was backcrossed as a nonrecurrent parent with Koshihikari as a recurrent parent. (b) Frequency distribution of heading date in the BC_3_F_2_ (Koshihikari×3//(Koshihikari/Isehikari F_2_). In the BC_3_F_2_, early maturing plants heading like Koshihikari during 8/12–8/17, midmaturing plants heading during 8/19–8/23, and late-maturing plants heading during 8/26–8/28 segregated in a ratio of 12 early maturations : 28 midmaturations : 10 late maturations, which was well consistent with a theoretical single gene ratio of 1 : 2 : 1 (*χ*^2^ = 0.88, 0.50 < *p* < 0.70). The semidwarfness derived from Isehikari was segregated independently from late maturity in F_2_ and F_3_ (a). As the latest homozygote without semidwarfness was used as a nonrecurrent parent, the late maturity was solely segregated in the backcross F_2_ (b), and finally, a late-maturing isogenic Koshihikari was developed.

In the F_2_ of Koshihikari×Isehikari, late-mature individuals that matured later than Isehikari were segregated. Progeny tests were conducted on 70 randomly selected F_2_ plants by using self-propagated 70 F_3_ lines from each F_2_ plant. The F_2_ genotype was determined by examination of the distribution of the heading date and culm length in the F_3_ lines. Twenty-three F_3_ lines were fixed in late-maturation headings between 8/31 and 9/8, which derived from late-maturing F_2_ plants that head from 8/23 to 9/2. Namely, the progeny from late-maturing F_2_ plants was confirmed to be fixed to late-maturation homozygotes. Twenty-one F_3_ lines derived from early-maturing F_2_ plants were fixed late-maturation headings between 8/26 and 9/2, which derived from early maturing F_2_ plants that head from 8/14 to 8/20. Namely, the progeny from early maturing F_2_ plants were confirmed to be fixed with early maturation homozygotes. On the other hand, 26 F_3_ lines, which derived from midmaturing F_2_ plants that head 8/18–8/28, segregated in heading dates from 8/26 to 9/8 straddling their parents' heading dates. These segregating F_3_ lines were thought to be derived from heterozygous F_2_ plants. Thus, the genotype of the heading date was determined according to the ratio of 23 late-maturing homozygous lines : 26 heterozygous lines : 21 early maturing homozygous lines (Figure 1(a)), which fit to a theoretical single gene ratio of 1 : 2 : 1 (*χ*^2^ = 4.74, 0.05 < *p* < 0.10). These results suggested that Isehikari has a single late-maturing gene.

**Figure 2:**
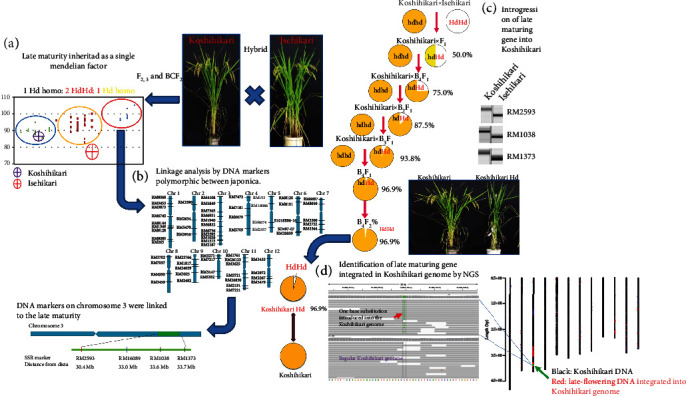
Genetic procedure to identify a candidate SNP for late maturity derived from a cultivar Isehikari. (a) Genetic analyses for late maturity through F_2_ and F_3_ between Koshihikari×Isehikari and the backcross BC_1~3_F_2_ by using Koshihikari as a recurrent parent with the latest F_3_ line in Koshihikari×Isehikari as a nonrecurrent parent. The late maturity derived from Isehikari was inherited as a single Mendelian factor. (b) Molecular linkage analysis by using late-maturing homozygous F_2_ segregants of Koshihikari×Isehikari by 66 SSR markers distributed on 12 rice chromosomes. The late-maturing gene was linked to the SSR markers on the long arm of chromosome 3. (c) Development of late-maturity isogenic Koshihikari by continuous backcrossing for NGS analysis. (d) Whole-genome analysis of isogeneic lines (BC_4_F_2_ and BC_6_F_2_) integrated with the late-maturing gene in the Koshihikari genome.

We then used the latest maturing F_3_ (confirmed late-maturing homozygote in F_3_, Figure 1(a)) as the first nonrecurrent parent for six times of continuous backcross with Koshihikari as a recurrent parent. BC_1_F_1_ was directly backcrossed with Koshihikari, and the most late-maturing segregant of BC_2_F_2_ was used for the third backcross with Koshihikari. In the BC_3_F_2_, early maturation-type plants heading like Koshihikari during 8/12–8/17, midmaturation-type plants heading during 8/19–8/23, and late-maturation-type plants heading during 8/26–8/28, which was 7 days later than Isehikari, were segregated as a histgram with 3 tops (Figure 1(b)). The segregation ratio was 12 early maturations : 28 midmaturations : 10 late maturations, which was well consistent with a theoretical single gene ratio of 1 : 2 : 1 (*χ*^2^ = 0.88, 0.50 < *p* < 0.70). BC_4_F_2_ was also segregated in a ratio of 8 early maturations : 11 midmaturations : 3 late maturations, which was well consistent with the theoretical 1: 2 : 1 ratio (*χ*^2^ = 2.27, 0.30 < *p* < 0.50). Thus, we identified a single major late-maturing gene derived from Isehikari, that is, incomplete dominance and that causes a 14-day delay flowering in the genetic background of Koshihikari derived from Isehikari. To map the late-maturing gene, a linkage analysis was conducted by using 23 F_2_ plants of Koshihikari×Isehikari by using 66 SSR markers distributed on 12 rice chromosomes, which showed polymorphism between Japonica cultivars Koshihikari and Isehikari. The results showed that the late-maturing gene was linked to the SSR markers RM2593, RM1038, and RM1373 on chromosome 3 (Figure 2(b)), with recombination values of 21.4, 11.7, and 20.2, respectively. Because this is a hybrid between Japonica cultivars, polymorphic DNA markers were limited and roughly linked. Then, we completed six times of continuous backcrossing with Koshihikari, and finally, a late-maturing isogenic Koshihikari was developed up to BC_6_F_2_, which was introduced with the target late-maturing gene derived from Isehikari, for the next-generation sequencing survey.

### 3.2. Next-Generation Sequencing Analysis of Late-Maturing Isogenic Line

**Figure 3:**
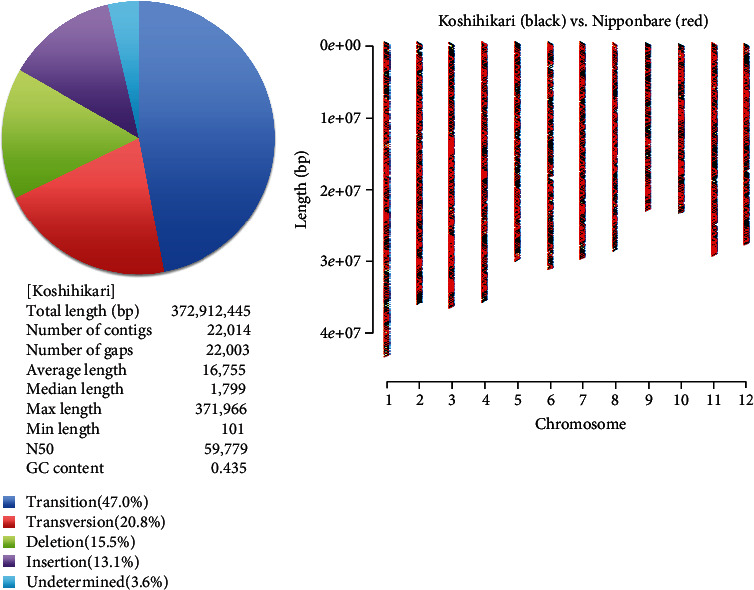
Construction of consensus genome of Koshihikari by whole-genome sequencing by next-generation sequencing. Read sequences gained from Koshihikari genome were mapped by using the Nipponbare genome as a reference sequence. As a result, a complete length bp of Koshihikari consensus sequence was determined with 99.0% cover ratio at the average depth of 35.68. Polymorphisms found among [Nipponbare] and [Koshihikari] contained 47.0 + 20.8 = 60.8% SNPs and 38.6% Indel.

**Figure 4:**
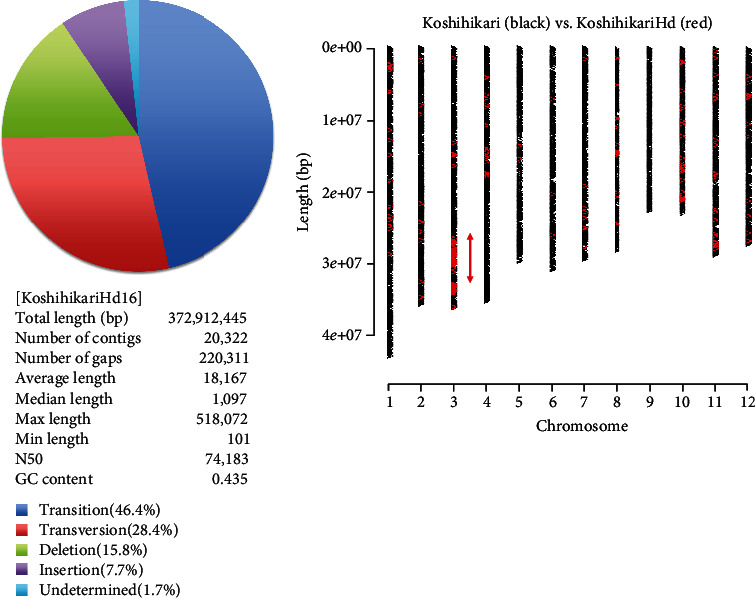
Whole-genome sequencing of late-maturing isogenic Koshihikari (BC_4_F_2_) by next-generation sequencer. Almost the whole sequence was substituted by Koshihikari (black): a large portion of the 12 rice chromosomes were substituted to the DNA sequence derived from Koshihikari (black), except for the block around the 33 Mb region in the long arm on chromosome 3 (red arrow) after continuous backcross targeting late-maturing gene. Namely, SNPs derived from Isehikari were concentrated and remained around the 33 Mb region of chromosome 3, which also showed a linkage relation with SSR markers (Figure 1). Polymorphisms among [Koshihikari Hd16] and [Koshihikari] contained 46.4 + 28.4 + 1.7 = 76.5% SNPs and 23.5% Indel.

**Figure 5:**
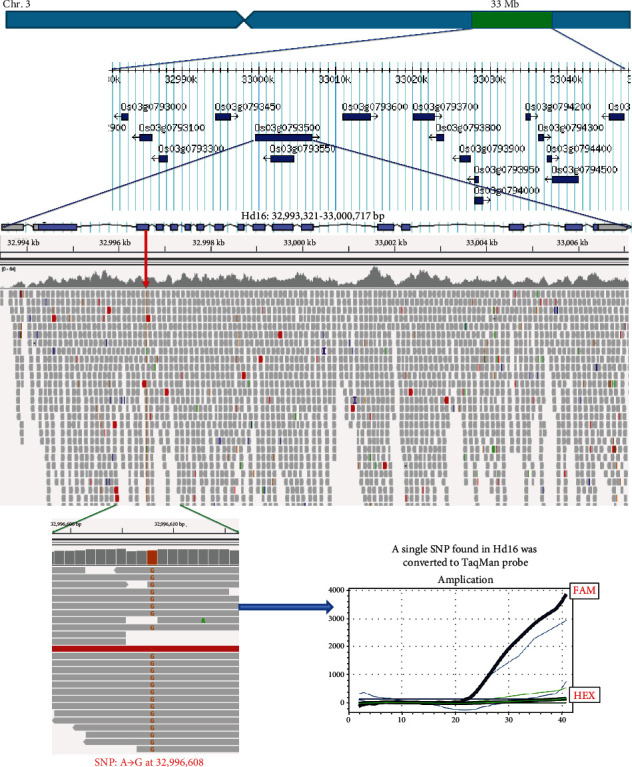
Identification of a single nucleotide substitution in *Hd16* (Os03g0793500) responsible for the late maturity of isogenic Koshihikari derived from Isehikari. In the region between linked SSR markers, only a single SNP in *Hd16* gene encoding casein kinase I was detected in several annotated coding sequences. The SNP was the same as that of *Hd16* gene in Nipponbare. TaqMan probe specific to *Hd16* allele labeled with FAM detected the SNP. The TaqMan probe for the SNP is useful for practical MAS breeding.

We conducted continuous backcrossing with Koshihikari to develop the late-maturing isogenic Koshihikari, which was introduced with the late-maturing gene of Isehikari into the Koshihikari genome. A whole-genome analysis by next-generation sequencing was conducted on Koshihikari and the late-maturation isogenic Koshihikari (BC_4_F_2_), which was integrated with the late-maturation gene derived from Isehikari. First, the read sequences gained by NGS from the Koshihikari genome were mapped by using the Nipponbare genome as a reference sequence. As a result, a 372,912,445 bp long consensus sequence of the Koshihikari genome was constructed at the average depth of 35.68 with 99.0% cover ratio (Table 1 and Figure 3). We then conducted a whole-genome sequencing of the late-maturing isogenic Koshihikari with high coverage (average 61.52). By using the consensus sequence of Koshihikari as a reference, read sequences gained from late-maturation isogenic Koshihikari were assembled at 99.1% coverage ratio with the average depth of 61.52 (Table 1), and SNP calling was conducted by using the vcf file. The results showed that a large portion of the 12 rice chromosomes were substituted to the same sequence as Koshihikari (black) (Figure 4), and SNPs and INDELs (red) derived from Isehikari were found to be concentrated around the vicinity of 33 Mb in the long arm of chromosome 3 (Figures 2 and 4). Namely, SNPs derived from Isehikari were found to be concentrated and remained within chromosome 3, which was also linked with SSR markers. Others were almost replaced by the Koshihikari genome. In the region linked to SSR markers, only a single SNP was detected in several annotated coding sequences (Figure 5). The SNP was situated in the *Hd16* gene (Os03g0793500) encoding casein kinase I, which was located at 32,993,321-33,000,717 (SNP : 32,996,608, A→G), and it caused a single amino acid change from threonine to alanine, at the distal end of the short arm of chromosome 3 in Koshihikari (Supplementary file 1). All 22 read sequences from the late-maturing isogenic Koshihikari showed the SNP (A→G at 32,996,608) in the *Hd16* gene (Os03g0793500) of Koshihikari. This result was reliable than sequencing of artificially amplified products by PCR. On the other hand, the DNA sequence of *Hd6* (Os03g0762000) in the late-maturing isogenic Koshihikari, which was also located at 31,496,180-31,490,533 from the distal end of the short arm of chromosome 3 near to *Hd16*, was completely identical to that of *Hd6* in the Koshihikari genome (Supplementary file 1). Therefore, the responsible gene for the late maturation of isogenic Koshihikari derived from Isehikari was identified as *Hd16* (Figure 5). DNA sequences of photoperiodic genes *Hd3a*, *RFT1*, and *Ehd1*, which are related with *Hd16*, in the late-maturing isogenic Koshihikari were confirmed to be identical to that of Koshihikari.

**Figure 6:**
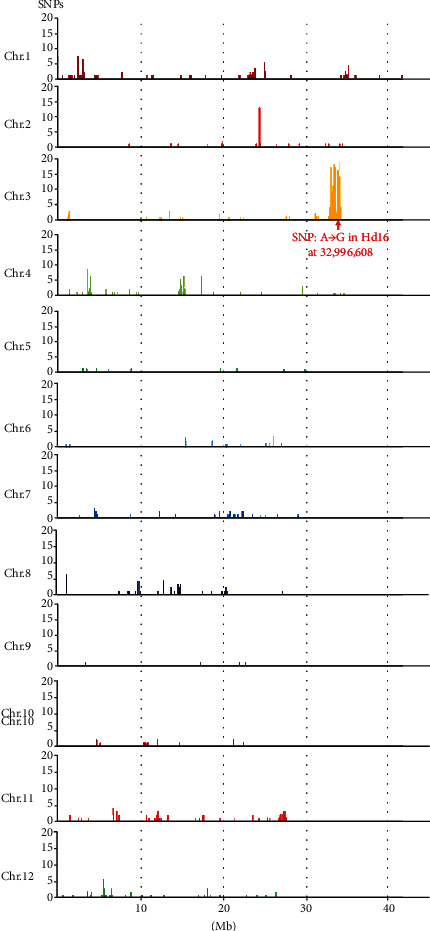
Frequency distribution of SNPs per 0.1 Mb in the genome of late-maturing isogenic Koshihikari (BC_6_F_2_) based on whole-genome sequencing. There were less than 10 SNPs per 0.1 Mb over all genomes, except for a SNP cluster around the 33 Mb region of chromosome 3 (yellow bar graph), which contained over 15 SNPs per 0.1 Mb. This SNP-concentrated region derived from Isehikari was also detected by linkage analysis with SSR markers (Figure 1). A large portion of the 12 chromosomes were substituted with the genome sequence of Koshihikari after 6 times of continuous backcross targeting a late-maturing gene.

The BC_4_F_2_ segregant that matured 15 days later than Koshihikari was used for the 5th backcross with Koshihikari. The resultant 35 BC_5_F_2_ plants were genotyped by using the SSR marker RM16089, which is closely linked to *Hd16* on chromosome 3. The ratio of [*hd16* homozygotes+heterozygotes] : *Hd16* homozygotes was 26 : 9, which was consistent with a 3 : 1 ratio (*χ*^2^ = 0.0095, 0.90 < *p* < 0.95). *Hd16* homozygous segregants from BC_5_F_2_ were selected and used for the 6th backcross to Koshihikari, and whole-genome sequencing analysis was conducted on *Hd16* homozygous late-maturing segregant from BC_6_F_2_, which was also confirmed by a TaqMan probe for the SNP in the exon 9 of *Hd16*. Read sequences gained from the late-maturing isogenic Koshihikari (BC_6_F_2_) were mapped to the consensus sequence of Koshihikari as a reference sequence. In total, 105,519,672 reads were mapped (mapped read rate of 99.92%) with a 19.52x genome coverage. Whole-genome sequencing detected a single nucleotide substitution (A→G) in the Koshihikari genome at the 32,996,608 bp position at the distal end of the short arm of chromosome 3 (Figures 5 and 6) the same as that in BC_4_F_2_.

## 4. Discussion

Flowering time of rice (*O. sativa* L.) is among the most important agronomic traits for regional adaptation and grain yield. Heading date is one of the most important traits in rice breeding, because it defines where rice can be cultivated and influences the expression of various agronomic traits. Rice, a facultative short-day plant, flowers early in short-day and late in long-day conditions. To date, more than 40 genes or quantitative trait loci (QTLs) controlling flowering time have been identified in rice, and diverse allelic variations for these flowering genes have been revealed [30]. In addition, rice flowers after a long vegetative growth period, during which flowering is inhibited by several independent pathways. For example, *Oryza sativa Phytochrome B* (*OsPhyB*), *Oryza sativa CONSTANS-like 4* (*OsCOL4*), *SUPERNUMERARY BRACT* (*SNB*), and *Oryza sativa INDETERMINATE SPIKELET 1* (*OsIDS1*) inhibit flowering regardless of day length. On the other hand, *grain number*, *plant height*, and *heading date 7* (*Ghd7*), *heading date 1* (*Hd1*), *heading date 5* (*Hd5*), *heading date 6* (*Hd6*), and *heading date 16* (*Hd16*) preferentially function to delay flowering under long-day conditions [31]. The *OsGI-heading date 1-* (*Hd1-*) *Hd3a/RICE FLOWERING LOUCUS T1 (RFT1)* pathway [32–34], which corresponds to the *GI-CO-FT* pathway common to all plants, including *Arabidopsis thaliana*, and the pathway particular to rice that includes *early heading date 1* (*Ehd1*), *Ghd7*, *Ehd2*, *Ehd3*, and *Ehd4* have been proposed as photoperiod-dependent flowering pathways for rice [35–37]. Under short-day conditions, the transcription of the FT gene *Hd3a* is induced via the independent genetic pathways of *Hd1* and *Ehd1* and the plant flower. The expression of *Hd1* regulated by the circadian clock gene *OsGI* induces the expression of *Hd3a*, after which flowering occurs [38]. However, *Ehd1*, the expression of which is induced via *Ehd2*, *Ehd3*, and *Ehd4*, promotes the expression of *Hd3a*/*RFT1* in both short-day and long-day conditions, and flowering is induced [33, 39–41]. *RFT1* is a paralog of the Florien gene *Hd3a*, in which 91% of the amino acid sequences are common [42]. However, the transcription activity of *Hd3a* is lower in long-day conditions than it is in short-day conditions, and flowering is suppressed. *Hd1* activates the expression of *Hd3a* in short-day conditions, but it suppresses the gene in long-day conditions. *Hd1* forms a complex with the photosensitive genes *Ghd7* and *DTH8*, suppressing *Ehd1* and *Hd3a/RFT1* and delaying flowering [32, 43, 44]. *Hd1* transcripts are phosphorylated by genes downstream of *Hd6*, which increases photosensitivity and causes late maturation. The expression of *RFT1* is promoted by *Ehd1* and *DTH2*, but it is suppressed via *Se14*. The expression of *Ehd1* is promoted via *Ehd2* and *Ehd4* but is suppressed via *DTH8* and *Ghd7*. The expression of *Ghd7* is induced by *Ehd3* and *Hd17*. *Ghd7* activity increases via phosphorylation by *Hd16*. Furthermore, *OsPRR37/Hd2* always suppresses blooming in both short-day and long-day conditions, independent of these transcription pathways [45]. Above findings indicated that the underlying regulation mechanism of flowering time in rice is very complicated. Moreover, the alteration of photoperiod sensitivity has let breeders diversify flowering time in rice and develop cultivars adjusted to a range of growing season periods.

In this study, we identified *Hd16* as a responsible gene for late maturation of Isehikari, which was discovered in the field of Ise Jingu Shrine. First, 149 F_2_ plants of Koshihikari×Isehikari and a total of 570 plants of 70 F_3_ lines self-propagated from 70 F_2_ plants were used for genetic analyses on heading date. The progeny test in F_3_ lines was in accordance with the theoretical single gene ratio of 1 late-maturing homozygous line : 2 heterozygous lines : 1 early maturing homozygous line. To map the late-maturation gene of Isehikari, a linkage analysis was conducted utilizing 66 SSR markers that distributed on 12 rice chromosomes that show polymorphism among Japonica rice. The late-maturation gene was mapped as a single Mendelian factor based on the linkage with the SSR markers located around 30–33 Mb on the short arm of chromosome 3. Furthermore, we developed a late-maturing isogenic line (BC_6_F_2_), which was integrated with the late-maturation gene of Isehikari in the genetic background of Koshihikari, via six times of continuous backcross. The existence of a single incomplete dominant late-maturation gene from Isehikari, which causes 14-day delay in maturation in the genetic background of Koshihikari, was identified. A next-generation sequencing analysis with high coverage was conducted, and 99.5% of the whole-genome sequence of Koshihikari and late-maturing isogenic Koshihikari were determined. Although the whole-genome sequence of Koshihikari has not been published, we have created a consensus sequence for Koshihikari. Finally, the cause of late maturation in Isehikari was identified as a single SNP in exon 9 of the *Hd16* gene in chromosome 3, which encodes casein kinase I.

The SNP found in the *Hd16* gene of Isehikari was the same as that of Nipponbare. *Hd16* of Nipponbare encodes a casein kinase I protein [36, 46]. Casein kinases (CKs) generally act as positive regulators in many signaling pathways in plants. In floral induction, *Hd16* acts upstream of the floral repressor *Ghd7*, and phosphorylates the transcripts of the photosensitive gene *Ghd7*, which is located upstream of the flowering gene *Ehd1*, in long-day conditions. It is thought to strengthen photosensitivity and delay maturation [36, 47]. The rice *Ghd7-Ehd1-Hd3a/RFT1* pathway modulated by *Hd16* is not present in *Arabidopsis* [37]. In this study, one nonsynonymous substitution in *Hd16* with Koshihikari allele resulted in decreased photoperiod sensitivity and a function to delay flowering time. On the other hand, *Hd6* encoding CK2, which is located at 31.5 Mb on chromosome 3, phosphorylates the transcripts of *Hd1* under long-day conditions and increases photosensitivity to suppress flowering [48]. *DTH8*, which is located at 4.3 Mb on chromosome 8 and encodes the NF-Yb subunit of the trimeric NF-Y transcription factor, forms a complex with *Hd1*, suppressing the expression of *Ehd1* and delaying blooming [43, 49, 50]. In this study, gene diagnosis by DNA resequencing showed that the photoperiodic genes *Ghd7*, *Ehd1*, *Hd3a*, *RFT1*, *Hd6*, and *DTH8* in the late-maturing isogenic Koshihikari were confirmed to be identical to those in Koshihikari.

Introgression of *Hd16*, *DTH2*, and *DTH8* into Koshihikari has been reported to cause a 10-day delay in maturation [36, 51–54]. In this study, we constructed a comprehensive isogenic genome via backcrossing, and we clarified the genome structure in which almost all sequences are substituted by the Koshihikari genome except for the vicinity around the *Hd16* gene on chromosome 3, by whole-genome sequencing. A 14-day delay of maturation is considered a noticeable effect of *Hd16* expression in the highly isogenic background. Fourteen-day late-maturing Koshihikari due to *Hd16* will avoid ripening in the high-temperature season in summer. Furthermore, the selection of a more regionally adaptive genotype is possible, instead of overuse of Koshihikari all over Japan. MAFF have registered the late-maturing isogenic Koshihikari integrated with *Hd16*, designated as “Koshihikari Suruga Hd16” as a new plant variety [55], which is a genetic resource to avoid heat damage when ripening during high-temperature summer periods by late maturation. Yield merit underpinning *Hd16* was also reported [56]. We also developed the late-maturing and semidwarf isogenic Koshihikari, which was integrated with both *Hd16* and semidwarf gene *d60* [57–59], designated as “Koshihikari Suruga d60Hd16” [60].

By using the methods described above, we conducted genetics-directed genomics approach through this study. We firstly genetically mapped the late-maturation gene utilizing a polymorphic population in early generations of a hybrid. Furthermore, we conducted genetic analyses in each BCF_2_ generation, which was aimed at integrating with a late-maturation gene through six times of continuous backcrossing with Koshihikari. Then, NGS analysis of the late-maturing isogenic Koshihikari identified only a single SNP in *Hd16* among the coding sequences in the 30–33 Mb region of the short arm of chromosome 3. This late-maturing Koshihikari with *Hd16* was registered as a new cultivar “Koshihikari Suruga Hd16” under Japanese varietal protection, which is useful to avoid flowering and maturity in the hottest August. The late-maturing Koshihikari is desired in the rice industry.

## 5. Conclusions

Genetic analyses of late maturity were conducted through the process of six times of continuous backcross with Koshihikari as a recurrent parent by using the late-maturing homozygous F_3_ line of Koshihikari×Isehikari as a nonrecurrent parent, thus developing a late-maturing isogenic Koshihikari (BC_6_F_2_). As a result, we identified a single late-maturing gene with incomplete dominance that caused the 14-day maturation delay of Koshihikari. The whole-genome sequencing was conducted on both of Koshihikari and the late-maturing isogenic Koshihikari. Finally, a single SNP was identified in the key gene *Hd16* of the late-maturing isogenic Koshihikari.

## Figures and Tables

**Table 1:** Summary of resequencing data by next-generation sequencing.

	Seq by Illumina HiSeq 4000		Mapping to the ref. genome		Mapping status			
Library name	Reads obtained (bp)	Bases generated (bp)	Reads unmapped/mapped ratio (%)	Ref. bases cover ratio (%)	*Q* median	*Q* mean	Depth median	Depth mean
Koshihikari_1	66,135,729	6,679,708,629	0.005884119	99.03155997	153	113.783	30	~35.67571
Koshihikari_2	66,135,729	6,679,708,629						
Koshihikari Hd-i_1	114,244,160	11,538,660,160	0.003406305	99.18284484	184	160.232	30	~61.51913
Koshihikari Hd-i_2	114,244,160	11,538,660,160						

A whole-genome analysis by next-generation sequencing was conducted on Koshihikari and the late-maturation isogenic Koshihikari (BC_4_F_2_), which was integrated with the late-maturation gene derived from Isehikari. First, the read sequences gained by NGS from Koshihikari genome were mapped by using the Nipponbare genome as a reference sequence. As a result, a 372,912,445 bp long consensus sequence of the Koshihikari genome was constructed at the average depth of 35.68 with 99.0% cover ratio. Next, by using the consensus sequence of Koshihikari as a reference, the read sequences gained from late-maturation isogenic Koshihikari were assembled at 99.1% cover ratio with the average depth of 61.52.

## Data Availability

The authors confirm that all the supporting data and protocols have been provided within the article. Sequence data that support the findings of this study are given in Supplementary file 1.

## Supplementary Materials

Supplementary Materials Supplementary file 1 shows the sequence data of *Hd16* and *Hd6*.
