# Supplementary material for: Whole-Genome Sequencing Revealed a Late-Maturing Isogenic Rice Koshihikari Integrated with Hd16 Gene Derived from an Ise Shrine Mutant
Source: Int J Genomics. 2022 Jan 6;2022:4565977. doi: 10.1155/2022/4565977 (PMC8758330; doi:10.1155/2022/4565977)
Supplement: Supplementary Materials — Supplementary file 1 shows the sequence data of Hd16 and Hd6. [file 4565977.f1.docx]

>*Hd6,* Os03g0762000 in Koshishikri=Sequence position= [chr03:31496180..31490533 (- strand)](https://rapdb.dna.affrc.go.jp/viewer/gbrowse/irgsp1?name=chr03:31508813..31514460)

CGTCCCACGC CGCCTCTATC TATCTCCACG TGAAATAAAA AAAAAACAAA GCTCCCGAAA ATATTCTCTC TCCCCCACCC CCGAAACCCT AGCGCGACCT CGCCGCCGGC AATGGCCGCA TGACCGATGC GCCTCCGCCG AGGAGCCGCC CGCACCCACC CAGCAGCAGC GTCGCCGTGC CCGCCGCCGC GGCGGCAGTG ATCGCAGCCG CCCTCGCGTC CTCCTTCCTC GCCCTGCTGC AGCCGCCCCG GCGCGCCCCG GTCGCCGCGG GATCCAGGGT CGGCATGTCG AAGGCGAGGG TCTACGCCGA CGTCAACGTG CTGCGCCCCA AGGAGTACTG GGACTACGAG GCGCTCACCG TTCAATGGGG GTAGGTAGCA CAGCCAGCCA GCTGACGTCA CCTTCCTGAG CCCCCTGATC AGCGGCCGTA GCTTGTATTC TCCAGATTTA GTTCGCGATC CGTATCCCGT ACACCTGGGC TGGGTTTGCT TATTGGGATT AGGTTGGATT ATTGGGTTAT GCGTAGGTTT GCTTGTGCCT GTAGATTTTG GTTTTGGTCA GGGAATTGGG AATTTATTGT AGCTTGAAGG TTAGATTGAA TTGCTTCTGT TTCTATTAGG ACGAACTCAA TACCGAAGAC TGCTTTGGTA GTTTTACATG TTTGTACTAT AGGAGTAGGG GACACATGTT TACCGAATGG TTGAAGAAAT TGTTATGAAT TTGCAAGGTT ATGATTTTAA TTTTGGAATC AATCTCACTA TATCTTCCTT TTAAAGTTGA TACTAGTGTT GTTCAGTTAA GAGCCTTTGT TTGATTGTGA ATGGCAAGCT GTAGGTATTG ATCCTATTTT TGTTGGGGAT AAAATCTAAG TTAAGGCAAA ATTAGGCAGT TTTATGTTTA ATCATTGGAA CAAAGTAAGT TGGTGATGGG TTTCTGGGTG TTTCTTTTGC ATCATCTGAT AACCAAGATT GATGAGTAAA GCATAACTTG GTAGTATAGT GCTTTGGGCC TAATCTTCTT TAGCACTGAA CATTCACCAA GTTCTATGCT TTTATGTAAT CTCAAATTTA ACATTGTGTT TTCCTTCACT CACCCTAGAA TATACTACCT GAAAGCAATC AATGAAATCA AATATAACTT CGTTTCTACC TATATGATTG TAACATGCTG AGTAATATGG TGCCAAACAA CTCAACACAT ATAATACTGT CCTTAACAAC CCATCTTCTT TTCCCTGTAG AAGTTACAGC CCTAGTATAT TCTGTACATG TCATGCTACC TAGATGACAG TTGAGGCCTG GTAGGAGTGT GCTTGTTTAA TTTTGGTACT CCAAAAGTGC ACTGTTTTTC TCAATCTGAC TCTGTTACCA GTTGTGTTTC CTCTAGATGT ATTCCTTATC TATGGTGAAT TATTAAATAA GTTGTCTGGT GACAAAAAAA AAAGAAAAAT AAAAGAAGAG ATGAACAATA TGTAGCTCAT TGATGATCCC TTGTCTGCTT GAACTTTATG AGAAACTATA GAAAGCAGTG GTGTTTTCCC TGACCTGATG TTAAATACTT GTTAAGAATT GAGCTTTCTT CGAAGTTTGT TCAGTTTACA CACCAACACT AAGAATTGCC ATATATCTCC CATCTTTTGT CCATTTAATT CTTGTTACCT CAAGTCATTG AGGGACCTGG CAGCATGTTA TGACTTACAC AATACCTCGC TAACTATTAT GGTGCATCTT TAACAGTGAG CAGGATGACT ATGAAGTTGT CAGGAAAGTT GGAAGAGGTA AATATAGTGA AGTCTTTGAA GGCATCAATG TTAACAACAA TGAGAAATGC ATCATCAAGA TACTCAAGCC TGTGAAGAAA AAGAAGGTAT TTAATTGATC TTATTGACTG TTTTTTTTAA TTGCTAGTGT TGAAGTTCTT AACCTACCTT TCATATGTTT GAACAGATCA AAAGGGAGAT TAAAATACTT CAGAATCTTT GTGGAGGTCC AAACATTGTG TAGCTTCTTG ATATTGTCAG AGATCAACAT TCTAAGACTC CTAGCTTGAT CTTTGAATAT GTCAACAATA CAGACTTCAA AGTGCTGTAC CCCACGTTGA CAGATTATGA TATCCGCTAC TACATATATG AGCTACTCAA GGTCTTCATT GAGCCTTCAT TGTCATCCCT ATTTATTTAC TCTATTCAGT AAAACATCCT GTTCTGTGGA TCTGTAGAAT GATGTATCTC TTATAGAAAT TGTTTCACAA TTACTTTCCT ATTATGTGAA GATCCAACTA AACACACTTG TAATATATCC TAGACAAATA TCACCATTCT CACTGCTTGC AAGTTGCAAC ATATCTTTAA TTATTTATGT ATATATGAAC TTGATTATTT TCTAAGTTAC ATGGCTTAAA ACTTGTCACA ATCTCAAGCA GTTTATGGAT CAGTTTTGTT TTGAGTTTTA ATTATAGTAG CATCTTGCAC TTCATAATGT ACAGATGACA AAAGAATTCC TGAATTGCAT ATGTGCTATA ATGGTTTATG ATCTGGGATT TTGAAGAGAA GTGTCGTTTT ATACATTTCT AAGTTCAGCA CTATGTTGGT GTTAAGAATT CAGCCATCAA TGGGCATCTT AACGTATGTG CTAGGTCATG CCTTCTATCC ATGGGTAATA AACTGTTAAC ACACAGTGTG TGTTTTTCAT ATCGATATTC TTAGCCAAGA ACAGTAGCAT CATTTGCCCT TAATCCTGTG TGTTAAGTTT GTTTAAAGAA TCTAGTTGAT TTTCTTTACA ATATTTTCCT TCTGTTTATG GCCCCAGGCA TTAGACTACT GCCATTCACA AGGCATTATG CATCGAGATG TCAAGCCCCA CAATGTTATG ATAGATCATG AGCTCCGAAA ACTTCGATTG ATAGACTGGG GCCTGGCTGA GTTCTATCAT CCAGGGAAGG AATATAATGT TCGTGTTGCT TCAAGGTTGG TGTAGTTACA AGCAAACTAC TTGTTTGGTT ATGATTTTCT TGCTTTTTTA TTGAATTGGA TTGCACCCTG ATAATCACTT GAATCATGAG AGGAAGCTAA CTTAAGAAGG TAGCATCCCT GTTTTGCAGT TTGTTTGCTA ACTTGGCTCT AGAAGCAATA CGTGAACCGA TAAATTACTT GGTTTGAATT CACTGCTACT GTTGAAGTCT GAATTGCCTA GTGGTCCTTT TGCAACATTA ATGTTACGAA ATGCTGAAAG TTAAGCAATG AAGCTGTTTA CCCTTAAACA ACTAAGTTTA CGTCTGAAAA AAAGGCAATA AAACAGATAC CATTACTAGC CCTTTATTAT TTTTGTAAGC ATGTTATCAC TGGAGTATAT CATGCAATTA TTGGGTGTAC GTCTGAAAAA AGGCAATAAA ACAGATACCA TTACTAGGAC TTTATTATTT TTGTAAGCAT GTTATCACTG GAATAGATCA TGCAATTATT GCTTACTAAT GCGTCAATTC TTTGCTCATT TTTGCTTTGG TACCTGAGTT GAGCATATGG TTTCTCGTTT TTATTCAGGT ATTTCAAGGG GCCTGAGCTT CTTGTTGATT TGCAAGATTA TGATTATTCT TTGGACATGT GGAGCCTTGG TTGCATGTTT GCTGGGATGG TATGTGTGGC TGTAAAAAAT ATCGCCTGTC TAGGTCAATG TCTGGATATC TAATGTACTA TTGTATTGAT AATAAGTCTG ACGTCTGAAC TCAGTTAACT GTATGCTATG ATGCAGATAT TCCGCAAGGA GCCATTCTTC TATGGTCATG ATAACCATGA TCAACTTGTC AAGATCGCAA AGGTAAGTCC CAGTTTGATT CTGGCCTCTC ACATTTCTCA AGGGAAAAAA AATGGTTTGG TATGCCTGAT AAAATGTTTA GTTATGCAAC TCGTGTTTTG GACTGGTTGG TATACATGTT TTACTTTGTT TCTAAAAAAA ATTGCTGTTT GTGCTCCTTT TAGCTTAGTA CTCATATGTT ATTCTGACAT ATAAGCAGTG TGATGTCGTC AAAATAAATT ATGTTCATTT GTAAATTGTG ATTTTTGAAG TTCTTATTTG TTGCTCTCGA ACTCTTACTA GGACGGTTAT TGGCATTTAA AGATGTTTTA AGCATCCAAT AATGCCTCGA GTGTGTGTCA GCAGTGTTGA TTCGCTTGTC ATCAGTTGAA AACTAAGTAC TTTTCCAGCA TTATGCTATT GATATCGGAC TAAGGCAGAT GTCATAATGT ACTTTGATAT CTATGCAAAT TTTATTCTTG ATCTGTTTTA GTGGTTTATA TAAGTGCTTA TTTTGGAATA ACAATAAAAC AGCTATATGT GAAATATTGG TATCTGATCC ATGTGTTTTC CCCATCATTC TCAGGTACTT GGAACAGAAG CACTAAATGC TTATTTGAAC AAGTACCATA TTGAGCTTGA TCCTCAGCTT GAAGCTCTTG TTGGGAGGTA CGTTGCCATG CTTTTAGATA TTGGTTTTGA ACGGGAAGAT TCAGAAGTAT AACACTTACA TATACATATG CAGGCATAGT AGAAAACCAT GGTCGAAATT CATTAATGCT GATAACCAAC ATCTAGTATC TCCTGAGGTT TGTCAATGGC TCTTGCTGTT TCCAAATCAA CCTTAAGATA ATGTTTGCTT AACATCATGC TTGTACATTT GTAGGCTGTA GATTTTCTTG ATAAGCTTCT ACGTTATGAT CACCAAGATA GGCTCACTGC ACGTGAAGCT ATGGTAAGTC TACCCCGACA GATAATATTT GTTACATTCC AAGAAGATAC TGATTTTGTT TGACTGGATA TTTCCTATTT ATGTAACAGT ATTGACTGTT CACTGAGATT GTTAGTTTAT TGCTGAATAT TTTAGTATAT ATCCTCCTTT TTAGTCATAA GAATTACATC AATGATGTCA TAATAGTACT TTCATCTTCC TATCCTATCA CACCTCTGTT CAATTTTTAT TTTAGGCATA TTCTGTTTCA CTTATTGCTC TGTATTATGA CAATATCATA AAACATTTTC CTGACCCTCA ACCAAAAATA GTTGGCAAGT TATGCATTTG TATAGGTACA CTTCAACTAG GGATGCAAGT GGAGCGGGCA ATCGGTTATT TTTTGCCTGT TTATCTCAAT TCTAGTTCAA TTGTTGTAGG TATTTATGCA GGTAACAGGA TTGCTTACTC GCATCGCTAA CTTCAACCCA ATATAATTTG GCAAATGGTG CATTTGGCAA TAGATAGAAA CCCTTCAAAT TTCTCTGCCA CATTGGCTTT TGTATGCAAT GAACAACGTT TCATCTTCAC ATAGTATCTG GCCAGTTGTA GGAGGAACAA ATTGTTATTT GATTACTCTT GGACTTCTCA AATTAATGCC ATAATCATGA ATACTTGCAG GCACATCCGT ACTTCCTCCA AGTGAGAGCT GCAGAAAATA GCAGAGCACG ACCACAATGA TCTTGTGTAC CTGCTAAAAT GATGATCCAG CTGATGATCC ACGACGGTAC TACTTTGAGT TTGTGTGAAC GATCGTGGAA TGTGCTTGTA GCCTTGCATT TGTAAACTGT AATTCACTCC GTTGGTTGCG TTTGATGAAT GCCGTGACAT GCACATAATT ATTTATTTCT GTAATGTTTT ACCATAAC

>*Hd6,* Os03g0762000 in the late maturing isogenic Koshishikri (BC_4_F_2_, BC_6_F_2_)=Sequence position=[chr03:31496180..31490533 (- strand)](https://rapdb.dna.affrc.go.jp/viewer/gbrowse/irgsp1?name=chr03:31508813..31514460)

CGTCCCACGC CGCCTCTATC TATCTCCACG TGAAATAAAA AAAAAACAAA GCTCCCGAAA ATATTCTCTC TCCCCCACCC CCGAAACCCT AGCGCGACCT CGCCGCCGGC AATGGCCGCA TGACCGATGC GCCTCCGCCG AGGAGCCGCC CGCACCCACC CAGCAGCAGC GTCGCCGTGC CCGCCGCCGC GGCGGCAGTG ATCGCAGCCG CCCTCGCGTC CTCCTTCCTC GCCCTGCTGC AGCCGCCCCG GCGCGCCCCG GTCGCCGCGG GATCCAGGGT CGGCATGTCG AAGGCGAGGG TCTACGCCGA CGTCAACGTG CTGCGCCCCA AGGAGTACTG GGACTACGAG GCGCTCACCG TTCAATGGGG GTAGGTAGCA CAGCCAGCCA GCTGACGTCA CCTTCCTGAG CCCCCTGATC AGCGGCCGTA GCTTGTATTC TCCAGATTTA GTTCGCGATC CGTATCCCGT ACACCTGGGC TGGGTTTGCT TATTGGGATT AGGTTGGATT ATTGGGTTAT GCGTAGGTTT GCTTGTGCCT GTAGATTTTG GTTTTGGTCA GGGAATTGGG AATTTATTGT AGCTTGAAGG TTAGATTGAA TTGCTTCTGT TTCTATTAGG ACGAACTCAA TACCGAAGAC TGCTTTGGTA GTTTTACATG TTTGTACTAT AGGAGTAGGG GACACATGTT TACCGAATGG TTGAAGAAAT TGTTATGAAT TTGCAAGGTT ATGATTTTAA TTTTGGAATC AATCTCACTA TATCTTCCTT TTAAAGTTGA TACTAGTGTT GTTCAGTTAA GAGCCTTTGT TTGATTGTGA ATGGCAAGCT GTAGGTATTG ATCCTATTTT TGTTGGGGAT AAAATCTAAG TTAAGGCAAA ATTAGGCAGT TTTATGTTTA ATCATTGGAA CAAAGTAAGT TGGTGATGGG TTTCTGGGTG TTTCTTTTGC ATCATCTGAT AACCAAGATT GATGAGTAAA GCATAACTTG GTAGTATAGT GCTTTGGGCC TAATCTTCTT TAGCACTGAA CATTCACCAA GTTCTATGCT TTTATGTAAT CTCAAATTTA ACATTGTGTT TTCCTTCACT CACCCTAGAA TATACTACCT GAAAGCAATC AATGAAATCA AATATAACTT CGTTTCTACC TATATGATTG TAACATGCTG AGTAATATGG TGCCAAACAA CTCAACACAT ATAATACTGT CCTTAACAAC CCATCTTCTT TTCCCTGTAG AAGTTACAGC CCTAGTATAT TCTGTACATG TCATGCTACC TAGATGACAG TTGAGGCCTG GTAGGAGTGT GCTTGTTTAA TTTTGGTACT CCAAAAGTGC ACTGTTTTTC TCAATCTGAC TCTGTTACCA GTTGTGTTTC CTCTAGATGT ATTCCTTATC TATGGTGAAT TATTAAATAA GTTGTCTGGT GACAAAAAAA AAAGAAAAAT AAAAGAAGAG ATGAACAATA TGTAGCTCAT TGATGATCCC TTGTCTGCTT GAACTTTATG AGAAACTATA GAAAGCAGTG GTGTTTTCCC TGACCTGATG TTAAATACTT GTTAAGAATT GAGCTTTCTT CGAAGTTTGT TCAGTTTACA CACCAACACT AAGAATTGCC ATATATCTCC CATCTTTTGT CCATTTAATT CTTGTTACCT CAAGTCATTG AGGGACCTGG CAGCATGTTA TGACTTACAC AATACCTCGC TAACTATTAT GGTGCATCTT TAACAGTGAG CAGGATGACT ATGAAGTTGT CAGGAAAGTT GGAAGAGGTA AATATAGTGA AGTCTTTGAA GGCATCAATG TTAACAACAA TGAGAAATGC ATCATCAAGATACTCAAGCC TGTGAAGAAA AAGAAGGTAT TTAATTGATC TTATTGACTG TTTTTTTTAA TTGCTAGTGT TGAAGTTCTT AACCTACCTT TCATATGTTT GAACAGATCA AAAGGGAGAT TAAAATACTT CAGAATCTTT GTGGAGGTCC AAACATTGTG TAGCTTCTTG ATATTGTCAG AGATCAACAT TCTAAGACTC CTAGCTTGAT CTTTGAATAT GTCAACAATA CAGACTTCAA AGTGCTGTAC CCCACGTTGA CAGATTATGA TATCCGCTAC TACATATATG AGCTACTCAA GGTCTTCATT GAGCCTTCAT TGTCATCCCT ATTTATTTAC TCTATTCAGT AAAACATCCT GTTCTGTGGA TCTGTAGAAT GATGTATCTC TTATAGAAAT TGTTTCACAA TTACTTTCCT ATTATGTGAA GATCCAACTA AACACACTTG TAATATATCC TAGACAAATA TCACCATTCT CACTGCTTGC AAGTTGCAAC ATATCTTTAA TTATTTATGT ATATATGAAC TTGATTATTT TCTAAGTTAC ATGGCTTAAA ACTTGTCACA ATCTCAAGCA GTTTATGGAT CAGTTTTGTT TTGAGTTTTA ATTATAGTAG CATCTTGCAC TTCATAATGT ACAGATGACA AAAGAATTCC TGAATTGCAT ATGTGCTATA ATGGTTTATG ATCTGGGATT TTGAAGAGAA GTGTCGTTTT ATACATTTCT AAGTTCAGCA CTATGTTGGT GTTAAGAATT CAGCCATCAA TGGGCATCTT AACGTATGTG CTAGGTCATG CCTTCTATCC ATGGGTAATA AACTGTTAAC ACACAGTGTG TGTTTTTCAT ATCGATATTC TTAGCCAAGA ACAGTAGCAT CATTTGCCCT TAATCCTGTG TGTTAAGTTT GTTTAAAGAA TCTAGTTGAT TTTCTTTACA ATATTTTCCT TCTGTTTATG GCCCCAGGCA TTAGACTACT GCCATTCACA AGGCATTATG CATCGAGATG TCAAGCCCCA CAATGTTATG ATAGATCATG AGCTCCGAAA ACTTCGATTG ATAGACTGGG GCCTGGCTGA GTTCTATCAT CCAGGGAAGG AATATAATGT TCGTGTTGCT TCAAGGTTGG TGTAGTTACA AGCAAACTAC TTGTTTGGTT ATGATTTTCT TGCTTTTTTA TTGAATTGGA TTGCACCCTG ATAATCACTT GAATCATGAG AGGAAGCTAA CTTAAGAAGG TAGCATCCCT GTTTTGCAGT TTGTTTGCTA ACTTGGCTCT AGAAGCAATA CGTGAACCGA TAAATTACTT GGTTTGAATT CACTGCTACT GTTGAAGTCT GAATTGCCTA GTGGTCCTTT TGCAACATTA ATGTTACGAA ATGCTGAAAG TTAAGCAATG AAGCTGTTTA CCCTTAAACA ACTAAGTTTA CGTCTGAAAA AAAGGCAATA AAACAGATAC CATTACTAGC CCTTTATTAT TTTTGTAAGC ATGTTATCAC TGGAGTATAT CATGCAATTA TTGGGTGTAC GTCTGAAAAA AGGCAATAAA ACAGATACCA TTACTAGGAC TTTATTATTT TTGTAAGCAT GTTATCACTG GAATAGATCA TGCAATTATT GCTTACTAAT GCGTCAATTC TTTGCTCATT TTTGCTTTGG TACCTGAGTT GAGCATATGG TTTCTCGTTT TTATTCAGGT ATTTCAAGGG GCCTGAGCTT CTTGTTGATT TGCAAGATTA TGATTATTCT TTGGACATGT GGAGCCTTGG TTGCATGTTT GCTGGGATGG TATGTGTGGC TGTAAAAAAT ATCGCCTGTC TAGGTCAATG TCTGGATATC TAATGTACTA TTGTATTGAT AATAAGTCTG ACGTCTGAAC TCAGTTAACT GTATGCTATG ATGCAGATAT TCCGCAAGGA GCCATTCTTC TATGGTCATG ATAACCATGA TCAACTTGTC AAGATCGCAA AGGTAAGTCC CAGTTTGATT CTGGCCTCTC ACATTTCTCA AGGGAAAAAA AATGGTTTGG TATGCCTGAT AAAATGTTTA GTTATGCAAC TCGTGTTTTG GACTGGTTGG TATACATGTT TTACTTTGTT TCTAAAAAAA ATTGCTGTTT GTGCTCCTTT TAGCTTAGTA CTCATATGTT ATTCTGACAT ATAAGCAGTG TGATGTCGTC AAAATAAATT ATGTTCATTT GTAAATTGTG ATTTTTGAAG TTCTTATTTG TTGCTCTCGA ACTCTTACTA GGACGGTTAT TGGCATTTAA AGATGTTTTA AGCATCCAAT AATGCCTCGA GTGTGTGTCA GCAGTGTTGA TTCGCTTGTC ATCAGTTGAA AACTAAGTAC TTTTCCAGCA TTATGCTATT GATATCGGAC TAAGGCAGAT GTCATAATGT ACTTTGATAT CTATGCAAAT TTTATTCTTG ATCTGTTTTA GTGGTTTATA TAAGTGCTTA TTTTGGAATA ACAATAAAAC AGCTATATGT GAAATATTGG TATCTGATCC ATGTGTTTTC CCCATCATTC TCAGGTACTT GGAACAGAAG CACTAAATGC TTATTTGAAC AAGTACCATA TTGAGCTTGA TCCTCAGCTT GAAGCTCTTG TTGGGAGGTA CGTTGCCATG CTTTTAGATA TTGGTTTTGA ACGGGAAGAT TCAGAAGTAT AACACTTACA TATACATATG CAGGCATAGT AGAAAACCAT GGTCGAAATT CATTAATGCT GATAACCAAC ATCTAGTATC TCCTGAGGTT TGTCAATGGC TCTTGCTGTT TCCAAATCAA CCTTAAGATA ATGTTTGCTT AACATCATGC TTGTACATTT GTAGGCTGTA GATTTTCTTG ATAAGCTTCT ACGTTATGAT CACCAAGATA GGCTCACTGC ACGTGAAGCT ATGGTAAGTC TACCCCGACA GATAATATTT GTTACATTCC AAGAAGATAC TGATTTTGTT TGACTGGATA TTTCCTATTT ATGTAACAGT ATTGACTGTT CACTGAGATT GTTAGTTTAT TGCTGAATAT TTTAGTATAT ATCCTCCTTT TTAGTCATAA GAATTACATC AATGATGTCA TAATAGTACT TTCATCTTCC TATCCTATCA CACCTCTGTT CAATTTTTAT TTTAGGCATA TTCTGTTTCA CTTATTGCTC TGTATTATGA CAATATCATA AAACATTTTC CTGACCCTCA ACCAAAAATA GTTGGCAAGT TATGCATTTG TATAGGTACA CTTCAACTAG GGATGCAAGT GGAGCGGGCA ATCGGTTATT TTTTGCCTGT TTATCTCAAT TCTAGTTCAA TTGTTGTAGG TATTTATGCA GGTAACAGGA TTGCTTACTC GCATCGCTAA CTTCAACCCA ATATAATTTG GCAAATGGTG CATTTGGCAA TAGATAGAAA CCCTTCAAAT TTCTCTGCCA CATTGGCTTT TGTATGCAAT GAACAACGTT TCATCTTCAC ATAGTATCTG GCCAGTTGTA GGAGGAACAA ATTGTTATTT GATTACTCTT GGACTTCTCA AATTAATGCC ATAATCATGA ATACTTGCAG GCACATCCGT ACTTCCTCCA AGTGAGAGCT GCAGAAAATA GCAGAGCACG ACCACAATGA TCTTGTGTAC CTGCTAAAAT GATGATCCAG CTGATGATCC ACGACGGTAC TACTTTGAGT TTGTGTGAAC GATCGTGGAA TGTGCTTGTA GCCTTGCATT TGTAAACTGT AATTCACTCC GTTGGTTGCG TTTGATGAAT GCCGTGACAT GCACATAATT ATTTATTTCT GTAATGTTTT ACCATAAC

>*hd16*, Os03g0793500 in Koshishikari=Sequence position=[chr03:32993321..33000717 (+ strand)](https://rapdb.dna.affrc.go.jp/viewer/gbrowse/irgsp1?name=chr03:32999502..33006898)

GCAGCAGCCG CAGCAGCAGC AAAGCCTCCC CCCCCAACCC ACCACCGCGA CACGCACGCG AGCGACGCGA GCGCTCCACG ATTTCTCTGC CTCCCCCCCC CCCTCGGTCT CCTCCTCCCC CACCACCACC GCACGCTTCC CGAGGCGCCG CCCTCCGCCG CCGCGGGCAT CCGCTTCCGA CGACGATGTA AGCCGCTCTT CTCCGCGCTC TCGCTCTGTG TTTTTTTTTT TTTTGGGGGG GGGGGTTGAT CGGTGGGGGC GGTTGCTTGA GGCGCGATCG GATTGCGGGG CGGGGTCGCT GACGCTGGGG GGGGAGTGAT CCGATGGGGT TGAGGAGGGC CGATTCGGGC GCTGGAAATG GGTTCGGTTT CGAGGATAGA TGCTTGCCTC ATGGGGAGCT TTATTGCGGA TTTAGGAAAA AAAGCGTGGT TTGCGTGGGA TTGGGGGATT TGTGGCGGGT TGGGATTAGT TTAGGATTGG TTTGGTGTTG GATTGGTGGT AATCTGGAGG TAAAAGGTGG GAGTTTTTTT TTTCCTTTCT GGGTGGAGAT CCCCTGCGGG GTATCGGGTG ATGGAGATGC TCGGGCTTCT TTTAGAGCGC CGATGGCGGC ATTCGATTCC GTTGAAAGGG CAGTGGTGGT AGCAAAGCGG GCTGCGTCTT TAAGGGGGGG GGGGGGGGGG CTCGCGATGG CGATGAATGC CTCAGATTGA TTCCACTCCG ATCTTTTCCT ATTCCCGATT CATTGCTTGA TTGATTTGCC ATGACATGGT GAAGGAACAA TCTTTGAGCT GAAATTTGTT TATGTTTGTG ATTGTTGTGC CTGCTGTGGT TTCCTTCATC AGTGATTTAT TCTTGTTGGA TGTTTTTTTT TCATGCGGAG ATTGGAAGGA GCACCGAAGG GTGTAGCACA AGGCCGAGTT GCATGGGGCG ATAATGCCAG AGTTGCGGGG TGGTGTTTGG AGAGCTCGTC TGAGGTCCAA GAAGGTTTAC GACGTCCAGG ACGCAGATCC AGCTGCGAGT CCGGTGTCGC CAGCTCCGCG GGGGAGGACT GGAAGGCGCG GCGGTGCTGC GGCTGGCAGA GGCAACAAGA CAGTTGCTGA GGGAGGAGGG AGGAAAGCTC TGAAGCCTAG AGGAAAGGGG TGCAGGGCTG TTGATTTGTG TAAGGATCAA CCTTGCAAGG ACCTCCCTGA AGTTATTGCT AGAAAGGCGG TTACCGGCAA AGCCCAGGAG GACCTTGGTT TGAACAAGGT AGCTGACAGA GCTGCGAACT TGATGATGGA CGGCGAAAGT GGAGACAAAT TTGCTGCGGC AGAAGATGAA TCCACTACAA CACCAGTTCC TGAGCGGGTA TGGAATACCT TCTTCCCTTT GCACTTCTGT AGGTGTACTA TCATTTGTAC CGATAAAGTC ATTTTCTTCA AGTTCTTTAA CATGGGATGT TTCATCATTT TTGTTTACTT TGTTTGGTAG TTTTTGTATG GTTGCTGTTC CAGATGTTCA TGTTACCTTT CACAGATGAC TTCTCTGCTG TATTGGTGTT ATTAGACACA AGACAACCGA CATGTCTGTT TCACATAAAA TATAGTGTAA CCAAATTTAG AAGTTATAAA GTATAATAAT TGATGATGAT TCATCTGAAT TGTGGCGTAA GAAATGGTTT AGTTGTTATG CTTGAGAGGT GAGTGCCTAT TTGCTTGGCG AAGCTGAGAT TGAGGCACTG AATGGCTGGA TTATAGTAGT TTTGTTTTGT AGAGGAAACA AACTTCAGAT AATCACATAG TATCACATCC TGATATCACA AAACCGATGA AACTCCGTGG CAAGTTCATC AAACAACAAA GTTTAGGAAT GGGAAAGTTA CAATCTCTTG CTCTTTGACA TCAAATTTTA TCACTAAAAT AGGAGTTTGC TTCTCCTGGT GACATCTTTT ATCTACACTT TATGTGGCAG AGTTTCACTG CCATTAATTA ACCTCCTAAA ATTCTAAATA GTTATATATG TTTTACAGGT TCAAGTAGGC AATTCCCCAG AGTATATAAC TGATAGGAAG TTGGGTAAAG GTGGATTTGG TCAGGTCTAT GTTGGTCGAA GAGTATCTGG TGGAGGTTCT CGCACGGGTC CAGATGCGCA AGAGGTTTCA TTTCTTACCA CCTCAATTGG ACACTGAAGC TCTGTTAGTT TAGTTAGTTT TTAGATATCT GAATGAATAT CCTGTAGGTT GCGCTTAAAT TTGAGCACCG AAGCAGTAAA GGATGTAACT ATGGCCCTCC ATACGAGTGG CAGGTTTACC AGTAAGAATT TCACACAATC TATCCCTTCT GATGTTTATG TTGTTCTATG TGGAGTATGA TCCATTAACC TTTTGTCCTT TTGTAAATAG CACTCTCAAT GGTTGTTATG GCATACCATC AGTCCACTAT AAGGGTCGTT TGGGAGACTA CTACATTCTT GTGCGTTTAC CTTAGTCATG TATTGAAAAA TGTTTCTGGA CTCAAAATAA ATTATTTTAT TCAACCATCA CACCAAAATT TCAGGTAATG GATATGCTTG GTCCCAGCCT CTGGGATGTG TGGAATTCAG TGGGACAGGC GTAAGTTTCG GATCACCTTT TTGCATTGAC CACCTGATTT TATTACTCCT CACTGAAAAT AACAGCATTG GAACTTGCAA ACAGGATGTC TGCCCATATG GTTGCTTGCA TTGCTGTGGA AGCGATATCA ATTCTTGAGA AGCTTCACTC TAAAGGGTAA AATTTAAAGT CACTTGTGCC TGAAAATTAT ATGGAGCTGC ATGGAATTTC TCTTTTGTTT TCTATTTCAT TGGTGTTAAC ATTTCCAGTG CATCTTATCA TTGCCAGGTT TGTACATGGT GATGTCAAAC CAGAGAATTT TTTGCTTGGT CATCCTGGGT CAGTTGATGA GAAGAAGCTT TTCCTGATTG ATCTTGGTTT AGGTAGGCTT TTGTTCAAAG TAGTCTCATC TGTGTATGAA GTATTCAATT TAGTTCTCAA TGTGCTATGG CATTTGCTCT TATGCATTTT GTACTTGAAT CCCAAGGAAT TGTTGCCCTG TTGATTTATT TTTATTTCGT GATGTTGAAA TCATGCAGCA TCCAGGTGGA AAGAAGCATC ATCTGGTCAG CATGTTGACT ATGATCAGAG GCCAGATGTC TTTAGGTTCG TCCATTTTTG TGTGTGCACT ATAATATCTT TTTCTCTGTA TTAAATTATG GGCAATTATT AACTTACCTT TTTCTTTTCG ACATTTTACA GGGGAACAAT TAGATACACT AGCGTCCATG CCCACTTAGG TCGTACAGGT AGCAGGAGGG ATGATTTAGA GTCACTGGCT TACACCCTAA TCTTTTTAAT AAGAGGGAGA TTACCTTGGC AAGGGTATCA GGTAGCCTTT AATATGCTCA GCAAAGTTTT GACATAATGT TTTCTTCTAT TTGTTGAACT TATTTTAGTG TTAATGGGAT GGTAATGCAG GGAGATAACA AGAGTTTTCT TGTTTGTAAG AAGAAAATGG CTACTTCACC AGAGTTGCTG TGTTGCTTCT GTCCAGCTCC GTTCAAACAT TTTCTAGAGA TGGTCACTAA CATGAAATTT GACGAAGAGC CAAACTATCC AAAACTTATT TCTCTCTTTG ATGGTTTGAT TGAAGGGCCT GCTTCAAGGC CCATCAGAAT TGATGGAGCT CTGAAGGTAT GTAAATGTTG TACACACAAC CTGTGTCTGT ACCATACAGA TTAACTTGAA TGCGTTAAAT TTTCTCATGT ATATTTTTGC ATATTATAGG TTGGGCAAAA ACGTGGAAGA ATGGTTGTAA ATCTTGACGA TGATGAACAG CCCAAGAAGA AAGTTAGGTT GGGAAGCCCA GCAACTCAAT GGATCTCAGT TTATAATGCT AGGCGGCCCA TGAAGCAGAG GTAATGCCCT AAAAACTGAA AATGGTGCAA AGTCTCTGTG TCTGTTATTT ATTTTCCCAA ACATTGTCCT CGCAATGCAC TTGCCTTTAA GTGTGGACGC ACTTGCCTTT AAGTGTGGAC TGAGCACATA ACACGATGCT ATCCAGTTGC CACCAGACAC TTTACAACTG GTCTGTTCTG TTTCCCTGAT GTCAGGATCT CATAAGGCTT CTTATCACTA TGATATTACG CAAACTGCTT TTGAGTTATC TCCGATACAT TTTATTGTTC TAACTACATT TTATATGTCT CCAAATTTGC TGGGTTAAGC AAGAGTTCAG TGACTAACTC TGTCTTGATT GATGTTGTCA CTGGGGATAG TTCCTGTATG CGCCTAGTGT CATCCATTTT AGCATGGACT TAACTTTACC TGATTCCTGG CAAAAGCAGC GATGTGAAAA TATCATCCAT GGCGTTAGCT GAACCCTTCA TGCTAGTTTT AGTGATGGAA GTGTTTGTGT GTGTGTGCGT GCCCCTGCAT GCCCATGCTT ATTACAATTT CAGCCTTCAT AGCTCTTATT TCTTTGGCCT CCTTGTTCTA GAATTTGAGT TTTTGAAGTC AACCTAGTTC TCAGCTATTA ATCCATATAT TTATCCTCTT TAAAAATTAA ATATGATATA TATTGACGTG GCTGCAAAAT GCATCACTTC TTTCTTTACA GATATCACTA TAATGTTGCT GATTCAAGGC TGCATCAGCA TATAGAAAAA GGCAATGAAG ATGGGCTGTA CATTAGTTGT GTCTCTTCTT CCGCAAATTT TTGGGCTCTC ATAATGGATG CTGGGACTGG CTTTTGTTCC CAAGTTTATG AGCTTTCACA AGTGTTTCTG CACAAGGTGC ACTGGCTATT TCTCCACCCC TTCCATCCAG TTTTGATGAA TCCAGAGTCC TGACTCCATA TTACCTTGTT TGTACATAGG ATTGGATTAT GGAGCAGTGG GAGAAGAATT ATTACATAAC GGCAATAGCA GGAGCAACCA ATGGAAGCTC ATTGGTTGTA ATGTCCAAAG GTTGGTAAAA TACCTTTTCT CTGCTGGCAA ACTAATTTTA CTACCTTGGC TATTTCTTAG AACTTGAAAC AACCCCGTTG ATGAATATGA AATAGTTGAA ACAGTCAGTT GCTTTAAGAC AGTTTTCAGC AGAACCTCTT GTGCAGCAAG GGTATGTGCC TTGCATACTA GACTGATTGA AACATGAGTG TGTTGAAGGC ATAACCTGCA ACAACTTCAT AGGTTAAAAT GATTTGTGTA GACTGTAGAG TGTGATCCTT TTTATTTGGG GGGACAGCCA CCAGTATTGC CTGCTCAATT

GCTCGGCAAG TAGCAATAAT TTTCGTTTCA TTGTGTAAAG GACGAACAAT CAGATGGTTC CTGTTAAAAC TGTGATGGTT AACAGTAGGT TTGTAAGTAG GCATTTCGTC CATGCTCTCA TCAGTCCATG GCAGCTTGAG GCAGAATTTA TTTAGAGGGT GCCACAAGTT CATAACATGA ATGGCAGACA GACATACATA ATCTGTCTCT ATAATACACA AATATCTATG AACTAATATT TGGTTTGGCC AGTTTTGATC AAAAAGTTGA TGGATCAATT TTGTTACCAG TTTTCCTTTG CTTCAACACT TAAATTTAAg agtaaatttc acaaaactac agatatattg accaaactat cacaaaactg cagatttaac accaaattta tcacaaaact acaggtctaa ggtggagtct cacaaaactt tagatttagt aacaaagttt tcacaaaact acaggtttag cgtcaattta atcacaaaac ttggacgttt tatgactcaa acataacatt agtgctaagg atttaaaccc caaaaattgt agttttgtga taattttata aatgtgtagt tttgtggtac ttagccttag acctgtaacc ttaaatctgt agttttgcat taatttggtc aaagtatctg tacttttgtg aaatttACTC TTTAATTTTG TATTTCTACT CTCAACTGTC TGGACTCCAA TTTTTCCTGT GAGCTTCTGG TGTCCTTCCA TTAGATACTA TCGTTCTAAA CCAGGCATGT ATCATCATTA CAGGAACTCC ATACACACAG CAGTCATACA AAGTCAGTGA ATCCTTTCCT TACAAGTGGA TTAACAAAAA GTGGAAAGAA GGTTTCCATG TGACATCTAT GGCTACTGCT GGAAACCGTT GGGGAGTTGT CATGTCAAGA AATGCAGGCT ATTCCCATCA GGTTAAAAGA TTTCGATTTA CCAATCCTTG TGTTCATTCT TTCTATGAAT TTGGTGGCTT CTCATATGCC AAATAACCTG TACGTGATAA AAAATGGTAT ATACACCTTG ATTGGTAAAG AATATCCAAC GATAAGTTAC TGTCGATAGG CTAACCTCAA AACCATCAGT TTAAGTGTTG CGTGCGGAAA CTACCTAACT CCAAACTTTT GTTGCTGTAA GGATGTCATC TTGTTTGTGG TTGAATTCGA ACATTCAACT GGTAGAATGC TTGATGTACC AATCAGTTGA GTTTGTGCTC TTTACAGCTG TTAACCAAAA AAGCAGGGAA ATACAGTAAC CACACATGTT TCTATTCTGA ATTTTCTTAC ATATTATCTT GATGTTCAGA CTCCTGAGTT GATCACTGCT TCATAGTGAG TAAGTCTGAA ACTGAATCCG TCTCGCAATG TGCAGGTGGT AGAGTTGGAC TTTCTATATC CAAGTGAAGG GATCCATCGG CGATGGGAGA CAGGTTACAG AATAACTTCG ACTGCAGCAA CTCCTGACCA AGCTGCCTTC ATCTTGAGCA TACCAAAGAG GAAGCCAATG GACGAGACAC AAGAAACTCT TCGAACTTCC TCCTTTCCCA GCAACCATGT CAAGGTACAT ACATCTTCAG CTCTCCCAAC CTCACAATGC CATACAAAAT CTTATGATGC GTCGAACTTT TTTTGACGGA AATGATGCGT CGAACTAACA TTGCTGTTAA AATCTCAGGA AAAATGGTCA AAGAACCTAT ACATCGCTTC AATCTGCTAC GGCCGGACCG TATGCTGACA GCTTCAACAT TTCGAAGCTC CAATGTGAAG CCATGTCTAG CTACTACTAG CATTTTGCAG TGAGCTGCCA ACCGATCAAA TCTCCCTTGA TTGGGAGATC TGAAGCAAGG AAAAAAAAAA GAAACAAATC AATGGCTGAC ATCACCGGCC TCTTGCAGCC ATCTATTCTA ACTGTAAAAA TTGAAAAAAA AAATCATGGC AAATGTTGTA CCAGATCGGA TATGGAGACC TTTGTCCATA AGCTAAGCTA GTATAAGCTG CATGGCGGTG CTAAGATCAT ATTGTTCTCA CATACGGAGT ATATATATAT CATACTACTC TCTCTgtttc agattataag atattttgac tttggcc

>*Hd16*, Os03g0793500 in the late maturing isogenic Koshishikri (BC_4_F_2_, BC_6_F_2_)=Sequence position=[chr03:32993321..33000717 (+ strand)](https://rapdb.dna.affrc.go.jp/viewer/gbrowse/irgsp1?name=chr03:32999502..33006898)

GCAGCAGCCG CAGCAGCAGC AAAGCCTCCC CCCCCAACCC ACCACCGCGA CACGCACGCG AGCGACGCGA GCGCTCCACG ATTTCTCTGC CTCCCCCCCC CCCTCGGTCT CCTCCTCCCC CACCACCACC GCACGCTTCC CGAGGCGCCG CCCTCCGCCG CCGCGGGCAT CCGCTTCCGA CGACGATGTA AGCCGCTCTT CTCCGCGCTC TCGCTCTGTG TTTTTTTTTT TTTTGGGGGG GGGGGTTGAT CGGTGGGGGC GGTTGCTTGA GGCGCGATCG GATTGCGGGG CGGGGTCGCT GACGCTGGGG GGGGAGTGAT CCGATGGGGT TGAGGAGGGC CGATTCGGGC GCTGGAAATG GGTTCGGTTT CGAGGATAGA TGCTTGCCTC ATGGGGAGCT TTATTGCGGA TTTAGGAAAA AAAGCGTGGT TTGCGTGGGA TTGGGGGATT TGTGGCGGGT TGGGATTAGT TTAGGATTGG TTTGGTGTTG GATTGGTGGT AATCTGGAGG TAAAAGGTGG GAGTTTTTTT TTTCCTTTCT GGGTGGAGAT CCCCTGCGGG GTATCGGGTG ATGGAGATGC TCGGGCTTCT TTTAGAGCGC CGATGGCGGC ATTCGATTCC GTTGAAAGGG CAGTGGTGGT AGCAAAGCGG GCTGCGTCTT TAAGGGGGGG GGGGGGGGGG CTCGCGATGG CGATGAATGC CTCAGATTGA TTCCACTCCG ATCTTTTCCT ATTCCCGATT CATTGCTTGA TTGATTTGCC ATGACATGGT GAAGGAACAA TCTTTGAGCT GAAATTTGTT TATGTTTGTG ATTGTTGTGC CTGCTGTGGT TTCCTTCATC AGTGATTTAT TCTTGTTGGA TGTTTTTTTT TCATGCGGAG ATTGGAAGGA GCACCGAAGG GTGTAGCACA AGGCCGAGTT GCATGGGGCG ATAATGCCAG AGTTGCGGGG TGGTGTTTGG AGAGCTCGTC TGAGGTCCAA GAAGGTTTAC GACGTCCAGG ACGCAGATCC AGCTGCGAGT CCGGTGTCGC CAGCTCCGCG GGGGAGGACT GGAAGGCGCG GCGGTGCTGC GGCTGGCAGA GGCAACAAGA CAGTTGCTGA GGGAGGAGGG AGGAAAGCTC TGAAGCCTAG AGGAAAGGGG TGCAGGGCTG TTGATTTGTG TAAGGATCAA CCTTGCAAGG ACCTCCCTGA AGTTATTGCT AGAAAGGCGG TTACCGGCAA AGCCCAGGAG GACCTTGGTT TGAACAAGGT AGCTGACAGA GCTGCGAACT TGATGATGGA CGGCGAAAGT GGAGACAAAT TTGCTGCGGC AGAAGATGAA TCCACTACAA CACCAGTTCC TGAGCGGGTA TGGAATACCT TCTTCCCTTT GCACTTCTGT AGGTGTACTA TCATTTGTAC CGATAAAGTC ATTTTCTTCA AGTTCTTTAA CATGGGATGT TTCATCATTT TTGTTTACTT TGTTTGGTAG TTTTTGTATG GTTGCTGTTC CAGATGTTCA TGTTACCTTT CACAGATGAC TTCTCTGCTG TATTGGTGTT ATTAGACACA AGACAACCGA CATGTCTGTT TCACATAAAA TATAGTGTAA CCAAATTTAG AAGTTATAAA GTATAATAAT TGATGATGAT TCATCTGAAT TGTGGCGTAA GAAATGGTTT AGTTGTTATG CTTGAGAGGT GAGTGCCTAT TTGCTTGGCG AAGCTGAGAT TGAGGCACTG AATGGCTGGA TTATAGTAGT TTTGTTTTGT AGAGGAAACA AACTTCAGAT AATCACATAG TATCACATCC TGATATCACA AAACCGATGA AACTCCGTGG CAAGTTCATC AAACAACAAA GTTTAGGAAT GGGAAAGTTA CAATCTCTTG CTCTTTGACA TCAAATTTTA TCACTAAAAT AGGAGTTTGC TTCTCCTGGT GACATCTTTT ATCTACACTT TATGTGGCAG AGTTTCACTG CCATTAATTA ACCTCCTAAA ATTCTAAATA GTTATATATG TTTTACAGGT TCAAGTAGGC AATTCCCCAG AGTATATAAC TGATAGGAAG TTGGGTAAAG GTGGATTTGG TCAGGTCTAT GTTGGTCGAA GAGTATCTGG TGGAGGTTCT CGCACGGGTC CAGATGCGCA AGAGGTTTCA TTTCTTACCA CCTCAATTGG ACACTGAAGC TCTGTTAGTT TAGTTAGTTT TTAGATATCT GAATGAATAT CCTGTAGGTT GCGCTTAAAT TTGAGCACCG AAGCAGTAAA GGATGTAACT ATGGCCCTCC ATACGAGTGG CAGGTTTACC AGTAAGAATT TCACACAATC TATCCCTTCT GATGTTTATG TTGTTCTATG TGGAGTATGA TCCATTAACC TTTTGTCCTT TTGTAAATAG CACTCTCAAT GGTTGTTATG GCATACCATC AGTCCACTAT AAGGGTCGTT TGGGAGACTA CTACATTCTT GTGCGTTTAC CTTAGTCATG TATTGAAAAA TGTTTCTGGA CTCAAAATAA ATTATTTTAT TCAACCATCA CACCAAAATT TCAGGTAATG GATATGCTTG GTCCCAGCCT CTGGGATGTG TGGAATTCAG TGGGACAGGC GTAAGTTTCG GATCACCTTT TTGCATTGAC CACCTGATTT TATTACTCCT CACTGAAAAT AACAGCATTG GAACTTGCAA ACAGGATGTC TGCCCATATG GTTGCTTGCA TTGCTGTGGA AGCGATATCA ATTCTTGAGA AGCTTCACTC TAAAGGGTAA AATTTAAAGT CACTTGTGCC TGAAAATTAT ATGGAGCTGC ATGGAATTTC TCTTTTGTTT TCTATTTCAT TGGTGTTAAC ATTTCCAGTG CATCTTATCA TTGCCAGGTT TGTACATGGT GATGTCAAAC CAGAGAATTT TTTGCTTGGT CATCCTGGGT CAGTTGATGA GAAGAAGCTT TTCCTGATTG ATCTTGGTTT AGGTAGGCTT TTGTTCAAAG TAGTCTCATC TGTGTATGAA GTATTCAATT TAGTTCTCAA TGTGCTATGG CATTTGCTCT TATGCATTTT GTACTTGAAT CCCAAGGAAT TGTTGCCCTG TTGATTTATT TTTATTTCGT GATGTTGAAA TCATGCAGCA TCCAGGTGGA AAGAAGCATC ATCTGGTCAG CATGTTGACT ATGATCAGAG GCCAGATGTC TTTAGGTTCG TCCATTTTTG TGTGTGCACT ATAATATCTT TTTCTCTGTA TTAAATTATG GGCAATTATT AACTTACCTT TTTCTTTTCG ACATTTTACA GGGGAACAAT TAGATACGCT AGCGTCCATG CCCACTTAGG TCGTACAGGT AGCAGGAGGG ATGATTTAGA GTCACTGGCT TACACCCTAA TCTTTTTAAT AAGAGGGAGA TTACCTTGGC AAGGGTATCA GGTAGCCTTT AATATGCTCA GCAAAGTTTT GACATAATGT TTTCTTCTAT TTGTTGAACT TATTTTAGTG TTAATGGGAT GGTAATGCAG GGAGATAACA AGAGTTTTCT TGTTTGTAAG AAGAAAATGG CTACTTCACC AGAGTTGCTG TGTTGCTTCT GTCCAGCTCC GTTCAAACAT TTTCTAGAGA TGGTCACTAA CATGAAATTT GACGAAGAGC CAAACTATCC AAAACTTATT TCTCTCTTTG ATGGTTTGAT TGAAGGGCCT GCTTCAAGGC CCATCAGAAT TGATGGAGCT CTGAAGGTAT GTAAATGTTG TACACACAAC CTGTGTCTGT ACCATACAGA TTAACTTGAA TGCGTTAAAT TTTCTCATGT ATATTTTTGC ATATTATAGG TTGGGCAAAA ACGTGGAAGA ATGGTTGTAA ATCTTGACGA TGATGAACAG CCCAAGAAGA AAGTTAGGTT GGGAAGCCCA GCAACTCAAT GGATCTCAGT TTATAATGCT AGGCGGCCCA TGAAGCAGAG GTAATGCCCT AAAAACTGAA AATGGTGCAA AGTCTCTGTG TCTGTTATTT ATTTTCCCAA ACATTGTCCT CGCAATGCAC TTGCCTTTAA GTGTGGACGC ACTTGCCTTT AAGTGTGGAC TGAGCACATA ACACGATGCT ATCCAGTTGC CACCAGACAC TTTACAACTG GTCTGTTCTG TTTCCCTGAT GTCAGGATCT CATAAGGCTT CTTATCACTA TGATATTACG CAAACTGCTT TTGAGTTATC TCCGATACAT TTTATTGTTC TAACTACATT TTATATGTCT CCAAATTTGC TGGGTTAAGC AAGAGTTCAG TGACTAACTC TGTCTTGATT GATGTTGTCA CTGGGGATAG TTCCTGTATG CGCCTAGTGT CATCCATTTT AGCATGGACT TAACTTTACC TGATTCCTGG CAAAAGCAGC GATGTGAAAA TATCATCCAT GGCGTTAGCT GAACCCTTCA TGCTAGTTTT AGTGATGGAA GTGTTTGTGT GTGTGTGCGT GCCCCTGCAT GCCCATGCTT ATTACAATTT CAGCCTTCAT AGCTCTTATT TCTTTGGCCT CCTTGTTCTA GAATTTGAGT TTTTGAAGTC AACCTAGTTC TCAGCTATTA ATCCATATAT TTATCCTCTT TAAAAATTAA ATATGATATA TATTGACGTG GCTGCAAAAT GCATCACTTC TTTCTTTACA GATATCACTA TAATGTTGCT GATTCAAGGC TGCATCAGCA TATAGAAAAA GGCAATGAAG ATGGGCTGTA CATTAGTTGT GTCTCTTCTT CCGCAAATTT TTGGGCTCTC ATAATGGATG CTGGGACTGG CTTTTGTTCC CAAGTTTATG AGCTTTCACA AGTGTTTCTG CACAAGGTGC ACTGGCTATT TCTCCACCCC TTCCATCCAG TTTTGATGAA TCCAGAGTCC TGACTCCATA TTACCTTGTT TGTACATAGG ATTGGATTAT GGAGCAGTGG GAGAAGAATT ATTACATAAC GGCAATAGCA GGAGCAACCA ATGGAAGCTC ATTGGTTGTA ATGTCCAAAG GTTGGTAAAA TACCTTTTCT CTGCTGGCAA ACTAATTTTA CTACCTTGGC TATTTCTTAG AACTTGAAAC AACCCCGTTG ATGAATATGA AATAGTTGAA ACAGTCAGTT GCTTTAAGAC AGTTTTCAGC AGAACCTCTT GTGCAGCAAG GGTATGTGCC TTGCATACTA GACTGATTGA AACATGAGTG TGTTGAAGGC ATAACCTGCA ACAACTTCAT AGGTTAAAAT GATTTGTGTA GACTGTAGAG TGTGATCCTT TTTATTTGGG GGGACAGCCA CCAGTATTGC CTGCTCAATT

GCTCGGCAAG TAGCAATAAT TTTCGTTTCA TTGTGTAAAG GACGAACAAT CAGATGGTTC CTGTTAAAAC TGTGATGGTT AACAGTAGGT TTGTAAGTAG GCATTTCGTC CATGCTCTCA TCAGTCCATG GCAGCTTGAG GCAGAATTTA TTTAGAGGGT GCCACAAGTT CATAACATGA ATGGCAGACA GACATACATA ATCTGTCTCT ATAATACACA AATATCTATG AACTAATATT TGGTTTGGCC AGTTTTGATC AAAAAGTTGA TGGATCAATT TTGTTACCAG TTTTCCTTTG CTTCAACACT TAAATTTAAg agtaaatttc acaaaactac agatatattg accaaactat cacaaaactg cagatttaac accaaattta tcacaaaact acaggtctaa ggtggagtct cacaaaactt tagatttagt aacaaagttt tcacaaaact acaggtttag cgtcaattta atcacaaaac ttggacgttt tatgactcaa acataacatt agtgctaagg atttaaaccc caaaaattgt agttttgtga taattttata aatgtgtagt tttgtggtac ttagccttag acctgtaacc ttaaatctgt agttttgcat taatttggtc aaagtatctg tacttttgtg aaatttACTC TTTAATTTTG TATTTCTACT CTCAACTGTC TGGACTCCAA TTTTTCCTGT GAGCTTCTGG TGTCCTTCCA TTAGATACTA TCGTTCTAAA CCAGGCATGT ATCATCATTA CAGGAACTCC ATACACACAG CAGTCATACA AAGTCAGTGA ATCCTTTCCT TACAAGTGGA TTAACAAAAA GTGGAAAGAA GGTTTCCATG TGACATCTAT GGCTACTGCT GGAAACCGTT GGGGAGTTGT CATGTCAAGA AATGCAGGCT ATTCCCATCA GGTTAAAAGA TTTCGATTTA CCAATCCTTG TGTTCATTCT TTCTATGAAT TTGGTGGCTT CTCATATGCC AAATAACCTG TACGTGATAA AAAATGGTAT ATACACCTTG ATTGGTAAAG AATATCCAAC GATAAGTTAC TGTCGATAGG CTAACCTCAA AACCATCAGT TTAAGTGTTG CGTGCGGAAA CTACCTAACT CCAAACTTTT GTTGCTGTAA GGATGTCATC TTGTTTGTGG TTGAATTCGA ACATTCAACT GGTAGAATGC TTGATGTACC AATCAGTTGA GTTTGTGCTC TTTACAGCTG TTAACCAAAA AAGCAGGGAA ATACAGTAAC CACACATGTT TCTATTCTGA ATTTTCTTAC ATATTATCTT GATGTTCAGA CTCCTGAGTT GATCACTGCT TCATAGTGAG TAAGTCTGAA ACTGAATCCG TCTCGCAATG TGCAGGTGGT AGAGTTGGAC TTTCTATATC CAAGTGAAGG GATCCATCGG CGATGGGAGA CAGGTTACAG AATAACTTCG ACTGCAGCAA CTCCTGACCA AGCTGCCTTC ATCTTGAGCA TACCAAAGAG GAAGCCAATG GACGAGACAC AAGAAACTCT TCGAACTTCC TCCTTTCCCA GCAACCATGT CAAGGTACAT ACATCTTCAG CTCTCCCAAC CTCACAATGC CATACAAAAT CTTATGATGC GTCGAACTTT TTTTGACGGA AATGATGCGT CGAACTAACA TTGCTGTTAA AATCTCAGGA AAAATGGTCA AAGAACCTAT ACATCGCTTC AATCTGCTAC GGCCGGACCG TATGCTGACA GCTTCAACAT TTCGAAGCTC CAATGTGAAG CCATGTCTAG CTACTACTAG CATTTTGCAG TGAGCTGCCA ACCGATCAAA TCTCCCTTGA TTGGGAGATC TGAAGCAAGG AAAAAAAAAA GAAACAAATC AATGGCTGAC ATCACCGGCC TCTTGCAGCC ATCTATTCTA ACTGTAAAAA TTGAAAAAAA AAATCATGGC AAATGTTGTA CCAGATCGGA TATGGAGACC TTTGTCCATA AGCTAAGCTA GTATAAGCTG CATGGCGGTG CTAAGATCAT ATTGTTCTCA CATACGGAGT ATATATATAT CATACTACTC TCTCTgtttc agattataag atattttgac tttggcc
